# Atractylenolide III ameliorates Non-Alcoholic Fatty Liver Disease by activating Hepatic Adiponectin Receptor 1-Mediated AMPK Pathway

**DOI:** 10.7150/ijbs.68873

**Published:** 2022-01-31

**Authors:** Qian Li, Jia-Xin Tan, Yong He, Fang Bai, Shi-Wei Li, Yi-Wen Hou, Long-Shan Ji, Ya-Ting Gao, Xin Zhang, Zhen-Hua Zhou, Zhuo Yu, Miao Fang, Yue-Qiu Gao, Man Li

**Affiliations:** 1 Laboratory of Cellular Immunity, Shuguang Hospital, Affiliated to Shanghai University of Traditional Chinese Medicine, Shanghai, China.; 2 Shanghai Institute of Materia Medica, Chinese Academy of Sciences, Shanghai, China.; 3 School of Life Science and Technology and Shanghai Institute for Advanced Immunochemical Studies, Shanghai Tech University, Shanghai, China.; 4 Department of Hepatopathy, Shuguang Hospital, Affiliated to Shanghai University of Traditional Chinese Medicine, Shanghai, China.

**Keywords:** ATL III, AdipoR1, oxidative stress, inflammation, AMPK, SIRT1

## Abstract

**Background:** Nonalcoholic fatty liver disease (NAFLD) is the most frequent cause of chronic liver diseases worldwide. At present, there are no effective pharmacological therapies for NAFLD except lifestyle intervention-mediated weight loss. Atractylenolide III (ATL III), the major bioactive component found in Atractylode smacrocephala Koidz, has been shown to exert anti-oxidant, anti-tumor, anti-allergic response, anti-bacterial effects and cognitive protection. Here we investigate the therapeutic potential and underlying mechanisms of ATL III for the treatment of NAFLD.

**Methods:** Male C57BL/6J mice were fed a high-fat diet (HFD) and treated with ATL III. Lipid accumulation was analyzed by Oil Red O staining in liver tissues and free fatty acids (FFAs)-treated hepatocytes. AMP-activated protein (AMPK) and sirtuin 1(SIRT1) signaling pathways were inhibited by Compound C and EX527 *in vitro*, respectively. Small-interfering RNA (siRNA) was used to knockdown adiponectin receptor 1 (AdipoR1) expression in HepG2 cells.

**Results:** ATL III treatment ameliorated liver injury and hepatic lipid accumulation in the HFD-induced NAFLD mouse model as demonstrated by that ATL III administration significantly reduced serum levels of alanine aminotransferase, glutamic oxaloacetic transaminase, triglycerides, total cholesterol and low-density lipoprotein. Furthermore, treatment with ATL III alleviated hepatic oxidative stress, inflammation and fibrosis in the HFD feeding model. To study the underlying mechanisms, we performed Computer Aided Design assay and found that open-formed AdipoR1 and adiponectin receptor 2 were the potential receptors targeted by ATL III. Interestingly, HFD feeding or FFAs treatment only reduced hepatic AdipoR1 expression, while such reduction was abolished by ATL III administration. In addition, *in vitro* treatment with ATL III activated the AdipoR1 downstream AMPK /SIRT1 signaling pathway and reduced lipid deposition in HepG2 cells, which was diminished by silencing AdipoR1. Finally, inhibition of AMPK or SIRT1, the AdipoR1 downstream signaling, abolished the protective effects of ATL III on lipid deposition and oxidative stress in FFAs-treated HepG2 cells.

**Conclusion:** Our findings suggest that ATL III is a therapeutic drug for the treatment of NAFLD and such protective effect is mediated by activating hepatic AdipoR1-mediated AMPK/SIRT1 signaling pathway.

## Introduction

Liver disease is one of the most serious health problems 1, and nonalcoholic fatty liver disease (NAFLD) is the most common type of liver diseases. NAFLD is characterized with lipid accumulation without significant alcohol consumption, followed by liver inflammation (non-alcoholic steatohepatitis, NASH) and occurs mostly in patients who are obese and have the metabolic syndrome 2-4. Recently, the prevalence of NAFLD has increased dramatically over the last three decades to nearly 15%-30% in western countries 5. An updated meta-analysis focusing on Asian epidemiological studies reported an NAFLD prevalence of 30% and an incidence of 50.9 cases per 1,000 person-years 6. It has been projected that China will have the largest number of liver-related deaths from NAFLD by 2030 7. The criterion for diagnosis of NAFLD is the presence of more than 5% triglycerides (TG) stored in hepatocytes 8. Many potential risk factors of NAFLD have been identified, such as overweight or obesity, diabetes and insulin resistance. However, the mechanisms underlying the progression of NAFLD have not been completely elucidated and there are currently no effective pharmacological therapies for NAFLD.

AMP-activated protein (AMPK) is a key sensor of cellular energy homeostasis and is activated by increased cellular adenosine mono-phosphate (AMP) and adenosine di-phosphate (ADP) 9. Activation of AMPK results in a decrease in lipogenesis and an increase in fatty acid oxidation and lipolysis in adipocytes *in vitro* and *in vivo* 10. The ability of AMPK to respond to altered AMP levels is dependent upon liver kinase B1 (LKB1), which phosphorylates AMPK 11. AMPK exerts many functions via the regulation of sirtuin 1 (SIRT1), a protein that plays an important role in regulating cell energy metabolism, cell stress, and cell fate 12. AMPK enhances SIRT1 activity by increasing cellular nicotinamide adenine dinucleotide (NAD+) levels, further leading to the deacetylation and regulation of downstream SIRT1 targets such as peroxisome proliferator-activated receptor gamma coactivator-1α (PGC1α) 13. Adiponectin is the most abundant adipose-specific hormone and exerts its functions by binding to its specific receptors, adiponectin receptor protein 1 (AdipoR1) and adiponectin receptor protein 2 (AdipoR2). AdipoR1 is abundantly expressed in skeletal muscle and liver, whereas AdipoR2 is predominantly expressed in the liver. Accumulating evidence suggests that activation of AdipoR1 and AdipoR2 plays important roles in regulating glucose and lipid metabolism, inflammation and oxidative stress. In the liver, activation of AdipoR1 and AdipoR2 induces the activation of AMPK pathways and PPARα pathways, respectively, which makes the AdipoRs as a potential drug target for NAFLD therapy 14.

Up to now, there were no FDA proved pharmacological therapies for NAFLD except lifestyle intervention-mediated weight loss. Therefore, it is urgent to identify the new therapeutic targets and therapies for the treatment of NAFLD. In recent years, many herbs and phytochemicals have been used as complementary and alternative therapies for liver diseases 15, 16. For example, Atractylenolide III (ATL III) is a sesquiterpene lactone and is the major bioactive component found in Atractylode smacrocephala Koidz. ATL III is also found in other medicinal herbs, such as Codonopsis pilosula, Atractylodes lancea, and Chloranthus henryi Hemsl. ATL III has been demonstrated to exhibit a series of benefits, including anti-oxidant, anti-tumor, anti-allergic response, anti-bacterial effects, and cognitive protection 17-19. However, the effects of ATL III on NAFLD have not been explored.

In the current study, we examined the effects of ATL III on NAFLD in high-fat diet (HFD)-fed mice and free fatty acids (FFAs)-treated HepG2 cells. We found that AdipoR1 is the potential combination receptor with ATL III by using computer Aided Design assay. Then the impacts of ATL III on lipid accumulation and oxidation and the relevant mechanisms are investigated. Our study revealed that ATL III treatment effectively ameliorated NAFLD by regulating AdipoR1 mediated AMPK-SIRT1 signaling pathway, suggesting that ATL III has therapeutic potential for the treatment of NAFLD.

## Materials and methods

### Chemical reagent

Stock solutions of 20 mM oleic acid (OA, Sigma-Aldrich, USA) and 40 mM palmitic acid (PA, Sigma-Aldrich) prepared in culture medium containing 1% fatty-acid-free bovine serum albumin (BSA, Sigma-Aldrich) were conveniently diluted in culture medium to obtain the desired final concentrations. A FFAs mixture of 3 mM (OA and PA at the proportion of 2:1) was prepared as stock solution 20. The 200 μg/ml stock solution of ATL III (purity ≥ 99%, Tauto Biotech, Shanghai) was prepared in dimethyl sulfoxide (DMSO) and the final concentration of DMSO was less 0.1% in all *in vitro* experiments.

### Animals and administration of ATL III

Six-week old male C57BL/6J mice were used throughout our experiments. HFD (No. D12492) was purchased from Research Diets, Inc. (Middlesex County, NJ, USA). Mice were randomly divided into the following three experimental groups (each group n=12) (1) control diet group (CD): mice were fed normal chow diet and administrated normal saline by tail intravenous injection; (2) the HFD-fed induced NAFLD mice group (HFD+DMSO): mice were fed an HFD (contained 60% fat) for 16 weeks and administrated DMSO by tail intravenous injection; (3) the HFD-fed induced NAFLD mice group administrated with ATL III (HFD+ATLIII): mice maintained an HFD and were administrated 10 mg/kg ATLIII (purity ≥ 98%, St. Louis, MO, USA). The 40 mg/ml stock solution of ATL III (purity ≥ 99%, Tauto Biotech, Shanghai) was prepared in DMSO, and then the stock solution was diluted with 0.9% NaCl. The final concentration of ATL III was 1 mg/ml, and it was injected by tail vein. Mice in both HFD+DMSO and HFD+ATL III groups maintained an HFD for 16 weeks and were then treated with DMSO or ATL III every other day in the last 4 weeks.

All animal experiments were approved by the Committee of Animal Experimentation (No. PZSHUTCM210320005) and conducted at Shanghai university of Traditional Chinese Medicine in accordance with its Animal Experiment Guidelines.

### Histological Analysis

Liver tissues were fixed with formalin and then embedded in paraffin. All fixed tissues were sliced into 4-μm slices and stained by hematoxylin and eosin (HE). All slices were examined using a light microscope (Olympus, Tokyo, Japan). The Ballooning Degeneration Score and NAFLD Activity Score were evaluated 21.

### Biochemical Analysis

Serum, liver tissues and cells lysates were individually collected. The levels of ALT (alanine aminotransferase), AST (Glutamic oxaloacetic transaminase), TG, total cholesterol (TC), high-density lipoprotein (HDL), low-density lipoprotein (LDL), malondiadehyde (MDA), glutathione peroxidase (GSH-Px) and superoxide dismutase (SOD) were tested with commercial kits according to the manufacturer's instructions (Nanjing Jian Cheng Bioengineering Institute, China).

### Prediction of the potential targets of ATL III

To identify the potential targets of ATL III, a molecular docking based computational target fishing simulation was designed and performed in this study. The genes (proteins) which were involved into the AMPK signaling pathway released from KEGG database (https://www.genome.jp/kegg/) were collected, and the structures of these proteins were found from RCSB (https://www.rcsb.org/). All structures of selected proteins were prepared by Protein Preparation Wizard of Schrödinger2020-3 22, in which we added missing hydrogens and side chain, generated proper ionization state of residues in pH 7.0 ± 2.0, and performed structure minimization with Optimized Potentials for Liquid Simulations 3e (OPLS3e) force field 23. The native ligands and the water of these proteins were removed for further reverse docking. The compound structure of ATL III was downloaded from PubChem 24, after which it was prepared by LigPrep of Schrödinger2020-3. During the ligand preparation, the force field was set as OPLS3e 23, and the ionization state was generated by Epik at a pH of 7.0 ± 2.0 25. Then we performed docking simulations to test the binding between ATL III and these proteins with the Glide v8.8 of Schrödinger 26, 27. The standard precision mode and the OPLS2005 force field were used and other parameters were kept as default. Then, all the binding complexes of the candidate proteins with ATL III were collected and ranked together, and the top ranked complexes by docking score were selected for further analysis. Residues around ATL III in a range of 5Å were treated as flexible, and the optimization was performed in variable-dielectric generalized Born solvation model and OPLS4 force field.

### Western Blot Analysis

Protein extracts were extracted from liver tissues or HepG2 cells using the Protein Lysis Kit (Beyotime, China) and then separated on 12% SDS-PAGE gels. Then, gels were transferred to polyvinylidene difluoride (PVDF) membranes and PVDF membranes were incubated with primary antibodies against AdipoR1, AdipoR2 (Affinity Biosciences, China), AMPK, phosphorylated-AMPK (p-AMPK), LKB1, phospho-LKB1 (p-LKB1) (Cell Signaling Technology, MA, USA), SIRT1, nuclear factor erythroid-2-related factor 2 (Nrf2), carnitine palmitoyltransferase-1A (CPT1A), silent information regulator transcription 3 (SIRT3), PGC1α and GAPDH (Proteintech Group, USA) overnight at 4 °C. The PVDF membranes were washed and incubated with secondary antibodies at room temperature for 1h. Finally, Omni-ECL^TM^ Femto Light Chemiluminescence Kit (EpiZyme, China) was used to detect specific protein expression. The images were obtained using Chemi-Lumin One Ultra (Tanon, China).

### Cell Culture and FFAs-induced steatosis of HepG2 cells

The hepatocellular carcinoma cell line HepG2 was purchased from Cell bank of the representative culture preservation committee of the Chinese Academy of Sciences, China. HepG2 cells were cultured in Dulbecco's modified Eagle's medium (DMEM; Gibco, USA) supplemented with 10% fetal bovine serum (FBS, Gibco), 100 units/ml penicillin and 100 μg/ml streptomycin under a humidified atmosphere of 95% air and 5% CO2 at 37 °C. HepG2 cells were incubated with 1 mM FFAs to stimulate lipid accumulation for 24 h 28, and then were treated with ATL III (0-50 μg/ml) for 24h to evaluate its effects on lipid metabolism. The control group was treated with an equivalent volume of 1% BSA solution. The control and FFAs-treated groups were treated with the same volume of DMSO 29. In *in vivo* exeriments, 25μg/ml ATL III was used to explore the protective mechanism.

### Cell viability Assay

The cells (5×10^3^ cells/well) were seeded in 96-well plates and cultivated overnight, and then cells were incubated with different concentrations of FFAs (0-1.5 mM) or ATL III (0-200 μg/ml) for 24 h or 48 h. Next, the cell counting kit-8 (CCK-8, AbMole Bioscience, USA) solution was added into the culture medium (10 μl/well), and the cultures were incubated for 2 h at 37 °C. The absorbance was measured at 450 nm by Microplate Reader (Bio-Rad; Hercules, CA, USA).

### Oil Red O staining

Oil Red O staining was used to evaluate the intracellular lipid droplet levels. Frozen liver blocks were sectioned into 6-μm slices. The liver slices and fixed HepG2 cells were washed with PBS and stained by Oil Red O working solution (Oil Red O dye and diluent at a ratio of 5:2) for 30 min at 4 °C. Then the liver slices and cells were washed with 60% isopropyl alcohol to remove the excess dye, and washed with PBS. Finally, the stained liver slices and HepG2 cells were observed by the Olympus microscope (Olympus, Tokyo, Japan). To further quantify the intracellular lipid content, the images were analyzed using Image J software.

### Measurement of intracellular reactive oxygen species (ROS)

The level of ROS was detected using fluorescent probe 2',7'-dichloro-dihydro-fluorescein diacetate (DCFH-DA, Nanjing Jian Cheng Bioengineering Institute, China). Cells were collected and incubated with DCFH-DA for 30 min at 37 °C in the dark. The fluorescence intensity was measured using flow cytometry (BD FACSCalibur, America).

### Inhibitor experiments

Compound C is the selective and reversible AMPK inhibitor 30. EX-527 is the selective SIRT1 inhibitor. The HepG2 cells were seeded into 6-well plates (5×10^5^ cells/well) for 12 h, and then were pre-incubated with presence or absence Compound C (2 mM, AbMole Bioscience, USA) or EX 527 (10 μM, AbMole Bioscience, USA) for 1 h. Next, cells were treated with FFAs for 24 h, and were incubated with FFAs and ATL III for another 24 h 31, 32.

### Interruption of AdipoR1

SiRNAs were chemically synthesized, annealed and transfected into 60-70% confluent HepG2 cells using Lipofectamine 3000 (Thermo Fisher Scientific, American) 33. The sequence of the AdipoR1-targeting siRNAs was as follows: CAGCTTTCGTCCACTTCTA. Unrelated siRNA sequence was used as negative control. AdipoR1-targeting siRNAs and unrelated siRNA sequence were synthesized by Fubio Biomedical Technology Co., Ltd, China.

### Enzyme-linked immunosorbent assay (ELISA)

Hyaluronic acid (HA), laminin (LN), Collage type VI (IV-C) and procollagen type III (PC III) were fibrosis-relevant proteins. The serum levels of HA, LN, IV-C and PC III were tested by ELISA according to the manufacture's recommended protocols (Vazyme Biotech Co, Ltd, China).

### Statistical Analysis

Statistical analyses were performed using the SPSS software (version 17.0). Test of Homogeneity of variance was performed. When the variance was uniform, one-way ANOVA followed by Fisher's Least Significant Difference (LSD) test was performed to compare data between two groups from multiple groups. When the variance was not uniform, Kruskal-Wallis test was performed to compare multiple groups. If there were significant differences among multiple groups, Mann-Whiteney U was performed to compare median between two groups. Then the test standard (α=0.05) was corrected by using Bonferroni method. Results were represented as mean±SD. The tests were all two-tailed, and statistical significance was set as *P* <0.05.

## Results

### ATL III administration ameliorates liver steatosis in HFD-fed mice

**Figure 1:**
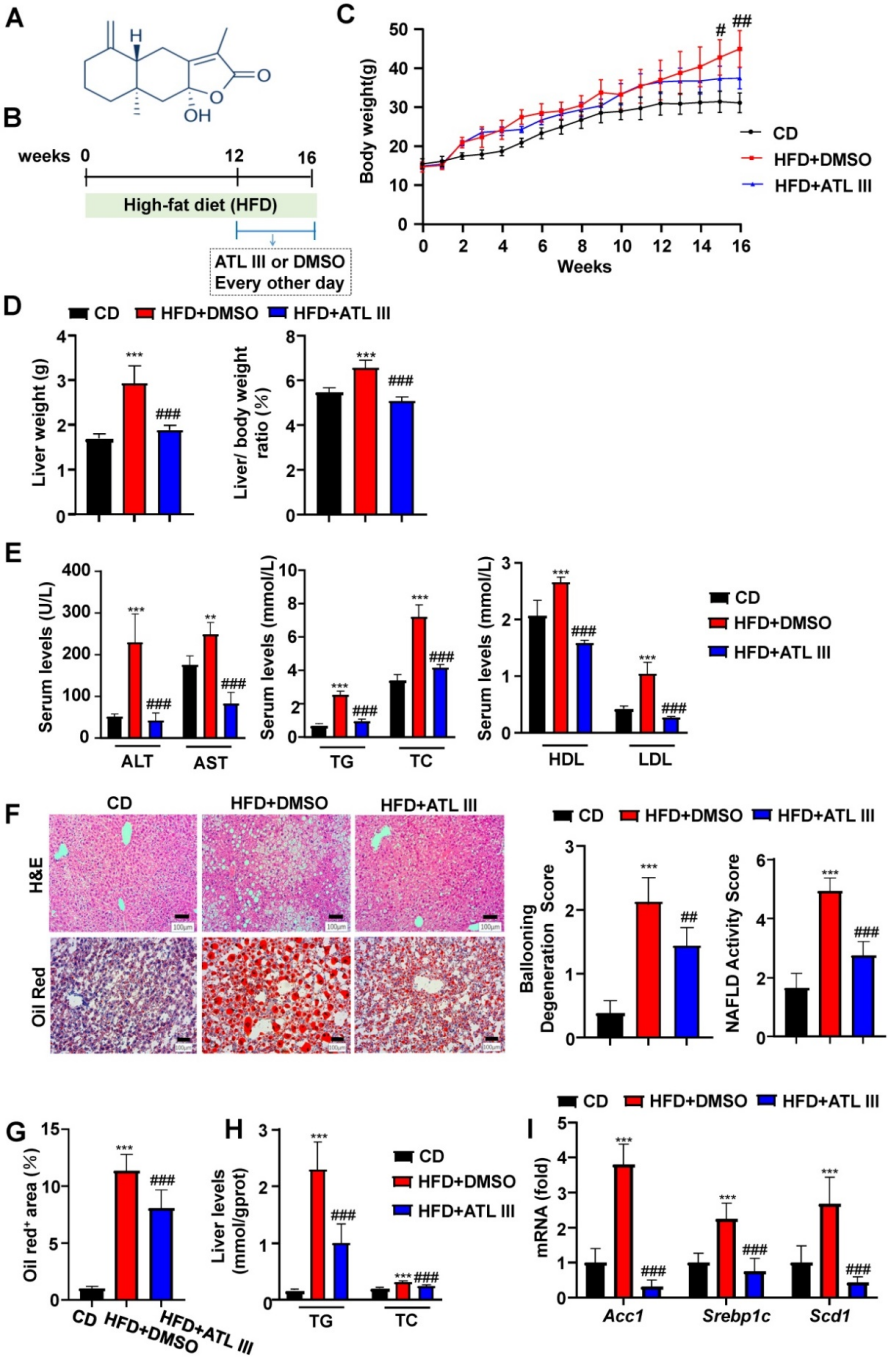
** ATL III administration ameliorates liver steatosis in HFD-fed mice.** Mice were categorized into three groups including control diet group (CD), the HFD-fed induced NAFLD group (HFD+DMSO) and the HFD-fed induced NAFLD group administrated with ATL III (HFD+ATL III). **A.** The structure of ATL III is shown. **B.** Mice were fed with an HFD for 16 weeks to induce NAFLD and administered DMSO or ATL III by tail intravenous injection in the last 4 weeks. **C.** Dynamic changes of weight during the experiment stage were recorded. **D.** Liver weight and liver/body weight ratio (%) were analyzed. **E.** Alanine aminotransferase (ALT), Aspartate aminotransferase (AST), Triglycerides (TG), Total Cholesterol (TC), High-density lipoprotein (HDL) and Low-density lipoprotein (LDL). Serum ALT, AST, TG, TC, HDL and LDL levels were tested. **F.** H&E and Oil Red O-stained slices of liver tissues were shown. Ballooning Degeneration Score and NAFLD Activity Score were calculated.** G**. The quantitation analyses of Oil Red O-stained slices of liver tissues were shown.** H.** TG and TC levels in liver tissues were examined. The 'mmol/gprot' means that the protein content of tissue extracts was calculated as mmols per gram protein of the tissue. **I.** The levels of some lipogenesis-related key genes including Acc1, Srebp1c and Scd1 in the livers of HFD-fed mice were tested by RT-qPCR. Values represent means±SD. (n=12 mice in each group). **, compared with CD *P*<0.01, ***, compared with CD *P*<0.001; #, compared with HFD+DMSO *P*<0.05; ##, compared with HFD+DMSO *P*<0.01, ###, compared with HFD+DMSO *P*<0.001.

ATL III is the main active constituent of Atractylodes rhizome, and its structure is shown in Figure 1A. To determine the protective effects of ATL III on NAFLD *in vivo*, male C57BL/6J mice were fed a HFD for 12 weeks followed by ATL III administration for 4 weeks, or DMSO as a control (Figure 1B). ATL III administration reduced mouse body weight (Figure 1C), but didn't affect food intake after HFD feeding (Supplementary Figure S1). As expected, ATL III-treated mice had less liver weight as compared with DMSO-treated mice after HFD feeding (Figure 1D). Furthermore, ATL III administration significantly ameliorated HFD-induced liver injury and lipid accumulation as evidenced by lower serum levels of ALT, AST, TG, TC and LDL in ATL III-treated mice (Figure 1E). Moreover, H&E and Oil red O staining showed that lipid accumulation, Ballooning Degeneration Score and NAFLD Activity Score in the liver were significantly decreased in ATL III-treated mice after HFD feeding compared with DMSO-treated mice (Figure 1F and 1G). Similarly, Liver TG and TC levels were also remarkably reduced in ATL III-treated mice after HFD feeding (Figure 1H). RT-qPCR analyses showed that hepatic up-regulation of some lipogenesis-related key genes including *Acc1*, *Srebp1c* and *Scd1* in HFD-fed mice was inhibited by ATL III treatment (Figure 1I). All the data strongly suggest that ATL III administration ameliorates HFD-induced liver injury and lipid accumulation.

### ATL III administration mitigates liver inflammation, fibrosis and oxidative stress in HFD-fed mice

**Figure 2:**
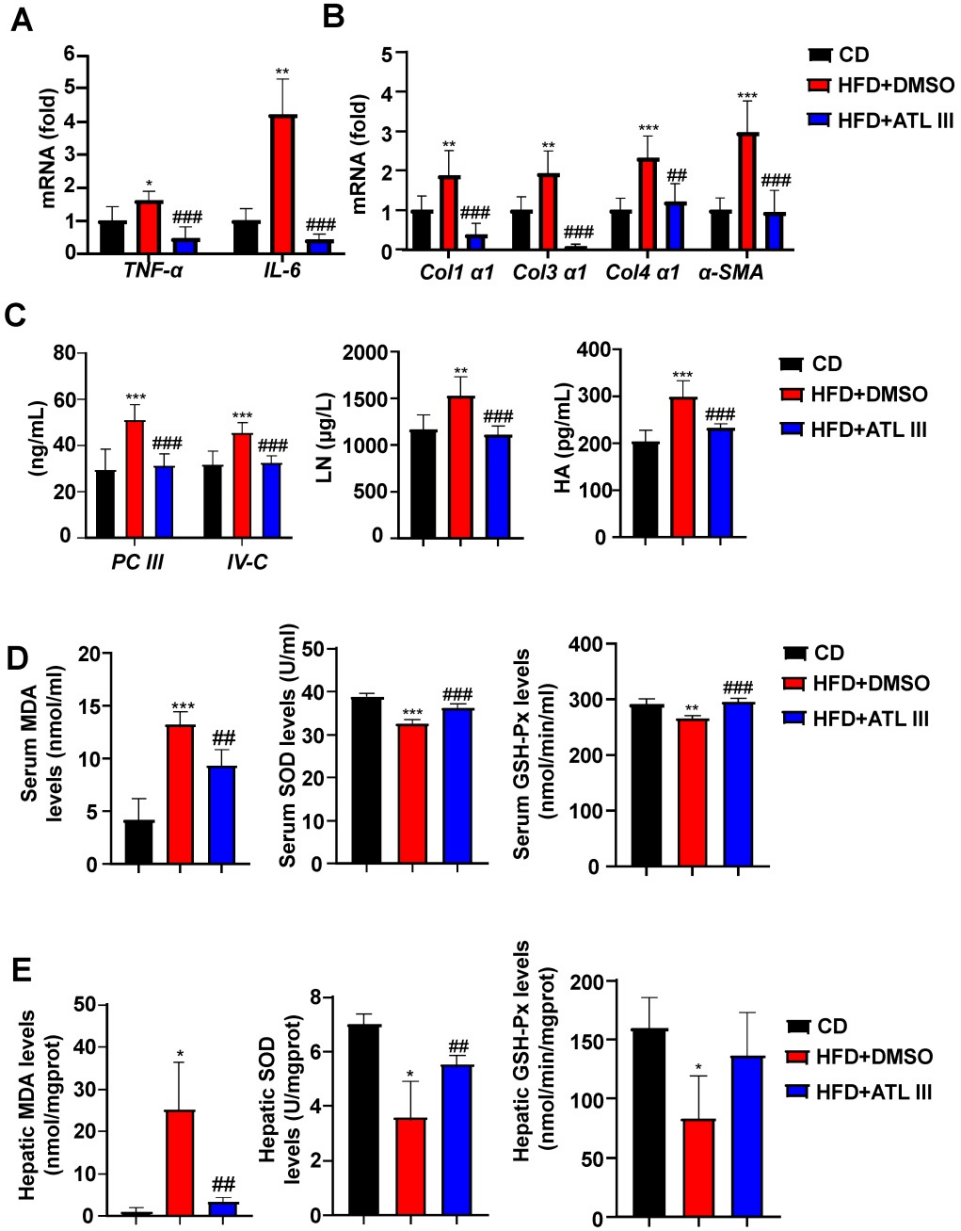
** ATL III administration mitigates liver inflammation, fibrosis, and oxidative stress after HFD feeding. A.** The levels of inflammatory factors including TNF-α and IL-6 in the livers of HFD-fed mice were tested by RT-qPCR. **B.** The levels of fibrogenic genes including Col1α1, Col3α1, Col4α1 and α-SMA were examined by RT-qPCR. **C.** Procollagen type III (PC III), Collage type VI (IV-C), Laminin (LN), Hyaluronic acid (HA). The expression levels of serum fibrosis-related proteins including PC III, IV-C, LN and HA were examined by ELISA. **D.** Malondialdehyde (MDA), Superoxide Dismutase (SOD), Glutathione peroxidase (GSH-Px). Serum MDA, SOD and GSH-Px levels were measured by chemocolorimetry. **E.** The expression levels of MDA, SOD and GSH-Px in liver tissues were examined by chemocolorimetry. The 'mgprot' mean that the protein content of tissue extracts and enzymatic activities were calculated as nmols and units per milligram protein of the tissue. Values represent means ± SD (n=12 mice in each group). *, Compared with CD *P*<0.05, **, compared with CD *P*<0.01, ***, compared with CD *P*<0.001; ##, compared with HFD+DMSO *P*<0.01, ###, compared with HFD+DMSO *P*<0.001.

Next, we examined whether ATL III ameliorated other NAFLD phenotypes. As illustrated in Figure 2A-C, ATL III injection decreased liver inflammation and fibrosis as demonstrated by that the levels of several inflammatory and fibrogenic genes in liver and fibrosis-relevant proteins in serum were significantly decreased in ATL III-treated HFD mice compared with DMSO-treated mice. In addition, we found that serum and hepatic MDA levels were dramatically elevated in HFD-fed mice compared with CD-fed mice, and such elevation was markedly reduced by ATL III treatment (Figure 2D and 2E). Importantly, serum levels of SOD and GSH-Px were significantly decreased after HFD feeding, and ATL III administration restored levels of SOD and GSH-Px (Figure 2D and 2E). Hepatic SOD and GSH-Px levels were markedly decreased after HFD feeding, but only the up-regulated levels of SOD induced by ATL III administration were significant.

### Incubation with ATL III reduces FFAs-induced lipid accumulation and ROS production in HepG2 cells

**Figure 3:**
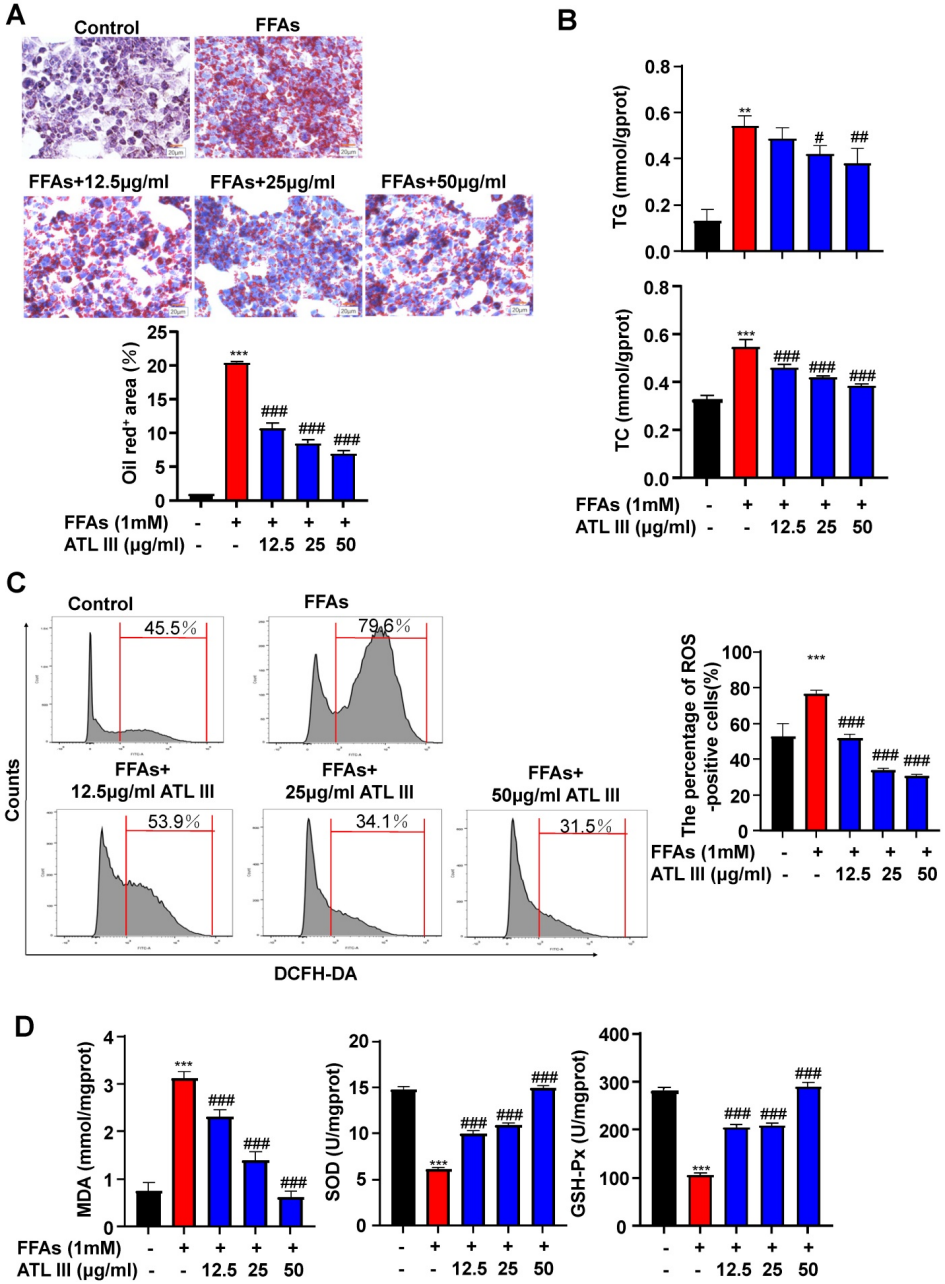
** ATL III administration reduces FFAs-induced lipid accumulation in HepG2 cells.** HepG2 cells were categorized into five groups including the control group, FFAs group, 12.5 µg/ml ATL III group, 25 µg/ml ATL III group and 50 µg/ml ATL III group. **A.** Lipids in HepG2 cells were stained by Oil Red O. Representative Oil Red O staining images were shown. The quantification of intracellular lipid content by Oil Red O staining was analyzed by calculating the area of intracellular lipid droplets. **B.** Intracellular TG and TC levels were measured by biochemical test kits. **C.** ROS expression levels in HepG2 cells were tested by flow cytometry. Representative flow cytometry analysis of ROS levels in HepG2 cells were shown. The analyses of ROS levels expressed by HepG2 cells were shown. **D.** MDA, SOD and GSH-Px levels expressed by HepG2 cells were measured. The above experiments were performed three times independently, and the results were displayed as mean±SD. **, Compared with Control group *P*<0.01 ***, compared with Control group *P*<0.001; #, compared with FFAs group *P*<0.05; ##, compared with FFAs group *P*<0.01; ###, compared with FFAs group *P*<0.001.

To investigate the molecular mechanisms by which ATL III inhibits lipid accumulation in hepatocytes, we treated HepG2 cells with different doses of FFAs and/or ATL III for 24h and 48h. Cell viability assay revealed that ATL III administration didn't affect hepatocyte viability (Supplementary Figure S2). FFAs treatment markedly increased fat content in hepatocytes, which was significantly reduced by ATL III administration in a dose-dependent manner (Figure 3A). ATL III treatment notably limited FFAs-induced accumulation of intracellular TG and TC in HepG2 cells (Figure 3B). In the murine hepatocyte cell line AML12, the fat content and intracellular TG and TC levels were increased by FFAs treatment, and the up-regulated levels induced by FFAs were all inhibited after ATL III treatment (Supplementary Figure S3).

To determine whether ATL III inhibited the oxidative stress in hepatocytes, we measured ROS, MDA, SOD and GSH-Px levels in HepG2 cells after FFAs treatment. As illustrated in Figure 3C, ROS levels were increased by treatment with FFAs while ATL III treatment inhibited FFAs-induced ROS production in HepG2 cells. In addition, the up-regulation of MDA levels induced by FFAs was blocked by ATL III administration. Next, we examined the anti-oxidant factors SOD and GSH-Px, and we found that FFAs treatment reduced SOD and GSH-Px levels in HepG2 cells, which was restored by ATL III incubation (Figure 3D).

### Computer Aided Design assay identifies AdipoR1 is a potential binding target of ATL III

**Figure 4:**
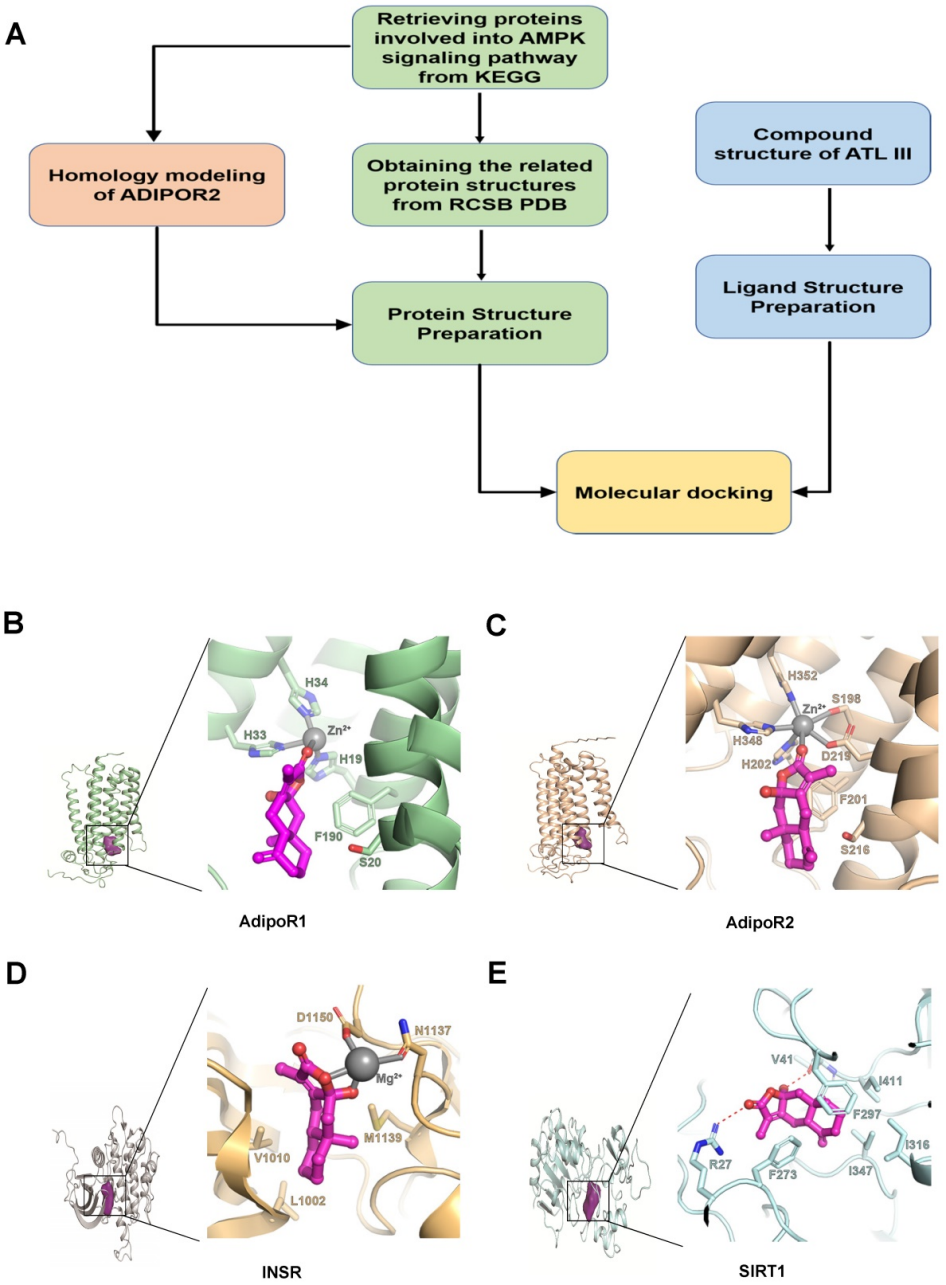
** The potential binding target of ATL III was identified by Computer Aided Design assay. A.** The designed workflow for identifying potential drug targets of ATL III from the genes being involved in the AMPK pathway was shown. **B.** AdipoR1: Zoomed potential binding pattern of AdipoR1 and ATL III. ATL III formed hydrophobic interaction with F190 and coordinate bond with zinc ion of AdipoR1 (GlideScore:-3.266). **C.** AdipoR2: Zoomed potential binding pattern of AdipoR2 and ATL III. ATL III formed hydrophobic interaction with F201 and coordinate bond with zinc ion of AdipoR2 as a part of hexa-coordinated complex (Glidescore:-3.478). **D.** INSR: Zoomed potential binding pattern of INSR and ATL III. ATL III formed two coordinate bond with magnesium ion and hydrophobic interaction with L1002, V1010 and M1139 of INSR (GlideScore; -3.478). **E.** SIRT1: Zoomed potential binding pattern of SIRT1 and ATL III. ATL III formed hydrogen bond with V41, R27 and stable hydrophobic interaction with five residues: F273, F297, I316, I347, I411 of SIRT1 (GlideScore:-5.969). GlideScore was the docking score for estimating the binding affinity between a ligand and a protein. In this work, the contribution of the metal ions to the binding affinity was not considered.

To understand the mechanisms by which ATL III ameliorates fat accumulation in hepatocytes, we searched ATL III targets by using Computer Aided Design assay, as shown in Figure 4A. The AMPK signaling pathway is well known to play an essential role in controlling the progression of NAFLD 34. Therefore, we focused on the genes being involved in this pathway to see if there is a candidate binding target of ATL III. As a result, 79 AMPK-related protein structures were obtained. All these protein structures were taken as our target candidates and their binding abilities with ATL III were computationally evaluated with molecular docking simulations. Through the calculations, four potential targets were identified, including open-formed AdipoR1, open-formed AdipoR2, Insulin Receptor (INSR) and SIRT1 (Figure 4B-E).

### HFD feeding down-regulates hepatic expression of AdipoR1 and its downstream signaling in mice, which is partially restored by ATL III treatment

**Figure 5:**
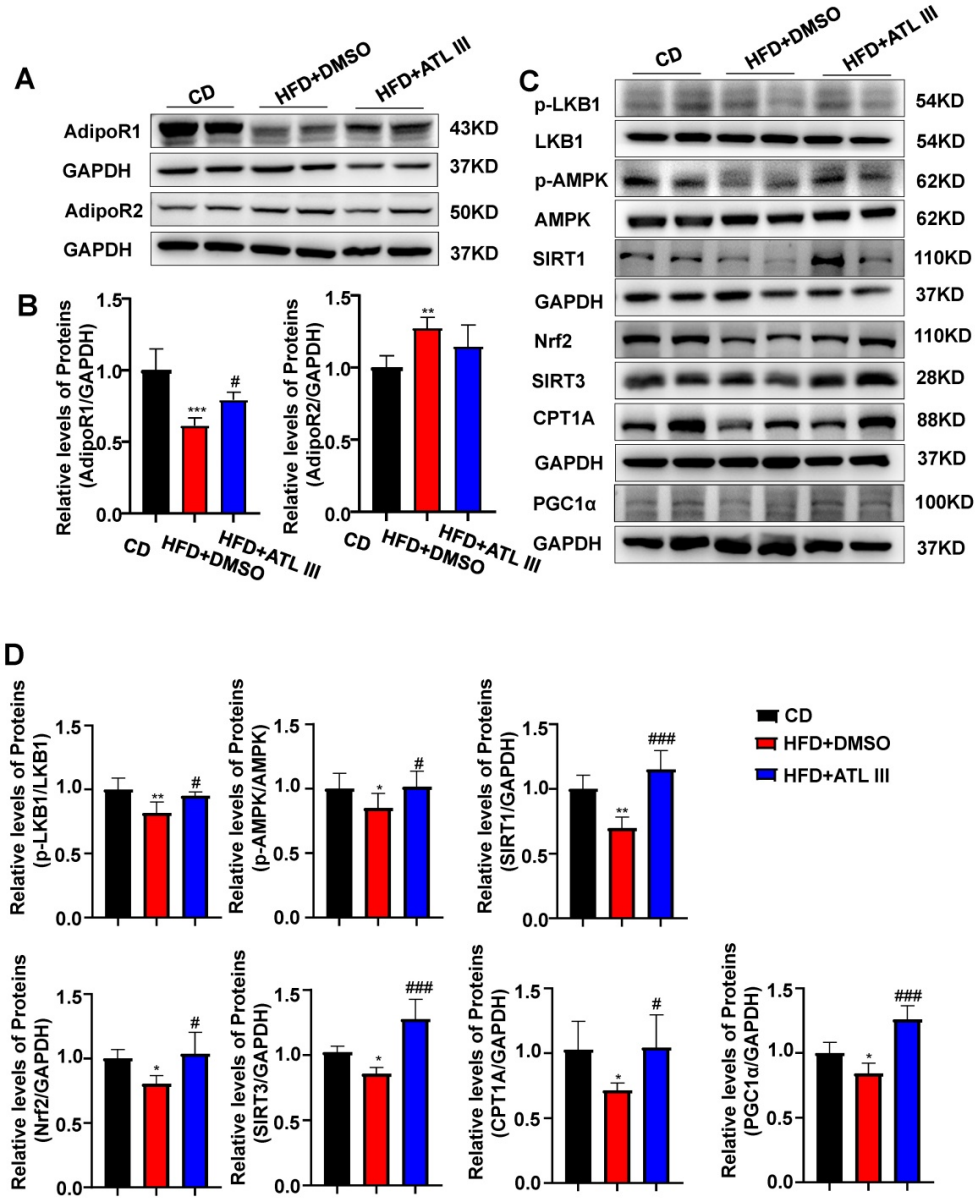
** ATL III treatment restores the down-regulation of hepatic AdipoR1 expression and AdipoR1 signaling induced by HFD feeding in mice. A.** Expression levels of hepatic AdipoR1 and AdipoR2 were examined in HFD mice. **B.** Relative levels of hepatic AdipoR1 and AdipoR2 were analyzed. **C.** AdipoR1 downstream signaling molecules including p-LKB1, p-AMPK, SIRT1, Nrf2, SIRT3, CPT1A and PGC1α were tested in HFD mice. **D.** Relative levels of p-LKB1, p-AMPK, SIRT1, Nrf2, SIRT3, CPT1A and PGC1α were analyzed. The experiments were performed three times independently, and values represent means ± SD.*, compared with CD *P*<0.05; **, compared with CD *P*<0.01; ***, compared with CD *P*<0.001; ^#^, compared with HFD+DMSO *P*<0.05; ^##^, compared with HFD+DMSO *P*<0.01; ^###^, compared with HFD+DMSO *P*<0.001.

Among the four potential ATL III targets, AdipoR1 and AdipoR2 are well known to regulate lipid metabolism in the pathogenesis of NAFLD 35. Then we examined hepatic expression of AdipoR1 and AdipoR2 in the HFD-induced NAFLD mouse model. As illustrated in Figure 5A, 5B and Supplementary Figure S4A, hepatic AdipoR1 levels were markedly decreased after HFD feeding while ATL III significantly restored hepatic AdipoR1 levels. However, hepatic AdipoR2 levels were increased in HFD-fed mice and showed a decreasing trend in ATL III-treated mice but didn't reach statistical significance. Most importantly, HFD feeding caused the down-regulation of AdipoR1-mediated downstream signaling including p-LKB1, p-AMPK, SIRT1, SIRT3, Nrf2, CPT1A and PGC1α protein levels, which were involved in lipid accumulation (LKB1 and AMPK), oxidative stress (SIRT3 and Nrf2) and fatty acid oxidation (CPT1A and PGC1α) 14. Such down-regulation was partially reversed by ATL III administration (Figure 5C, 5D and Supplementary Figure S4B).

### FFAs treatment down-regulates the expression of AdipoR1 and its downstream signaling in HepG2 cells, which is partially restored by ATL III treatment

**Figure 6:**
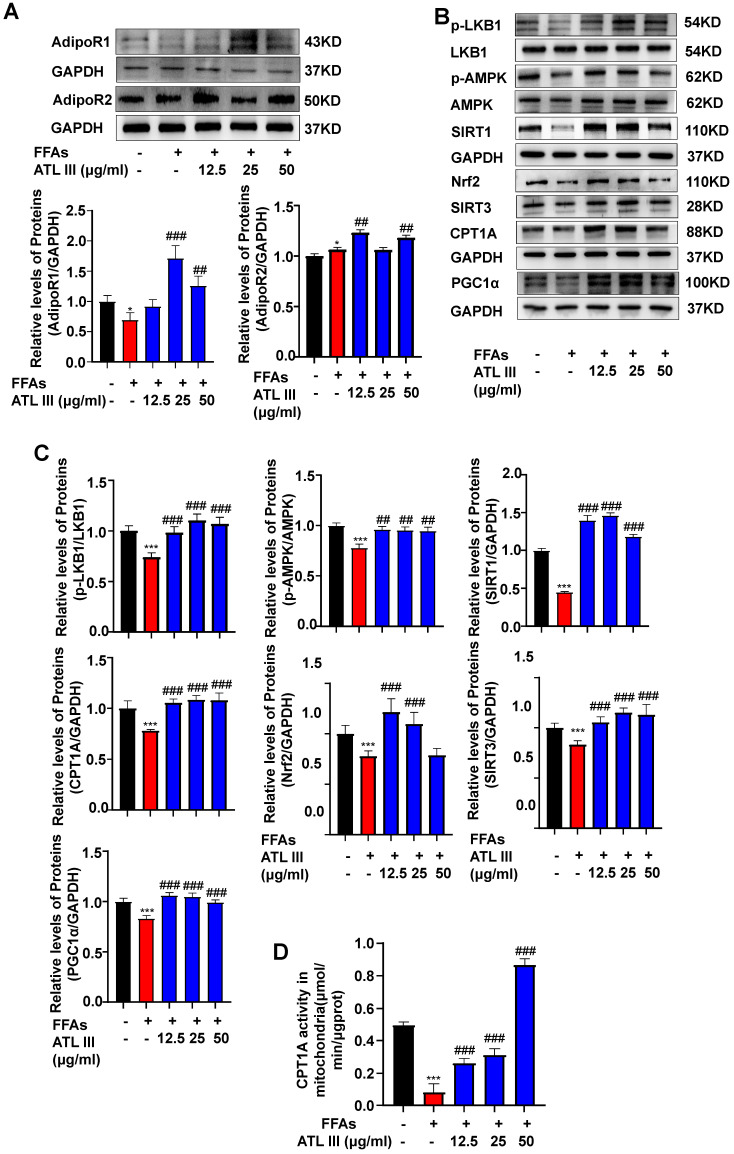
** ATL III restores the down-regulated expression of AdipoR1-mediated AMPK-SIRT1 signaling molecules in HepG2 cells. A.** HepG2 cells were categorized into five groups including the control group, FFAs group, 12.5 µg/ml ATL III group, 25 µg/ml ATL III group and 50 µg/ml ATL III group. AdipoR1 and AdipoR2 protein levels were examined by western-blot. Relative levels of hepatic AdipoR1 and AdipoR2 were analyzed**. B.** The expression levels of a number of key components of AdipoR1-mediated downstream signaling molecules were assessed by western blot. **C.** Relative levels of p-LKB1, p-AMPK, SIRT1, Nrf2, SIRT3, CPT1A and PGC1α were analyzed. **D.** The CPT1A activity was examined by using a CPT1A activity detection kit. The experiments were performed three times independently, and values represent means±SD. *, Compared with Control group *P*<0.05; ***, compared with Control group *P*<0.001; ^##^, compared with FFAs group *P*<0.01; ^###^, compared with FFAs group* P*<0.001.

To examine whether ATL III reverses FFAs-induced AdipoR1 down-regulation *in vitro*, HepG2 cells were directly treated with FFAs and/or ATL III. As illustrated in Figure 6A and Supplementary Figure S5A, FFAs treatment only reduced AdipoR1 protein levels in hepatocytes and such reduction was restored by ATL III treatment. FFAs treatment increased AdipoR2 levels, and such increase was strengthened by ATL III treatment, which suggested that AdioR2 maybe not involved in FFAs-induced steatosis of HepG2 cells. Furthermore, ATL III treatment partially abolished FFAs-induced down-regulation of AdipoR1-mediated signaling pathways (Figure 6B, 6C and Supplementary Figure S5B). In addition, we found that CPT1A activity was decreased by FFAs treatment, and such decrease was abolished by ATL III treatment (Figure 6D).

### Silencing AdipoR1 abolishes ATL III-mediated amelioration of lipid accumulation and AdipoR1 downstream signaling in HepG2 cells

**Figure 7:**
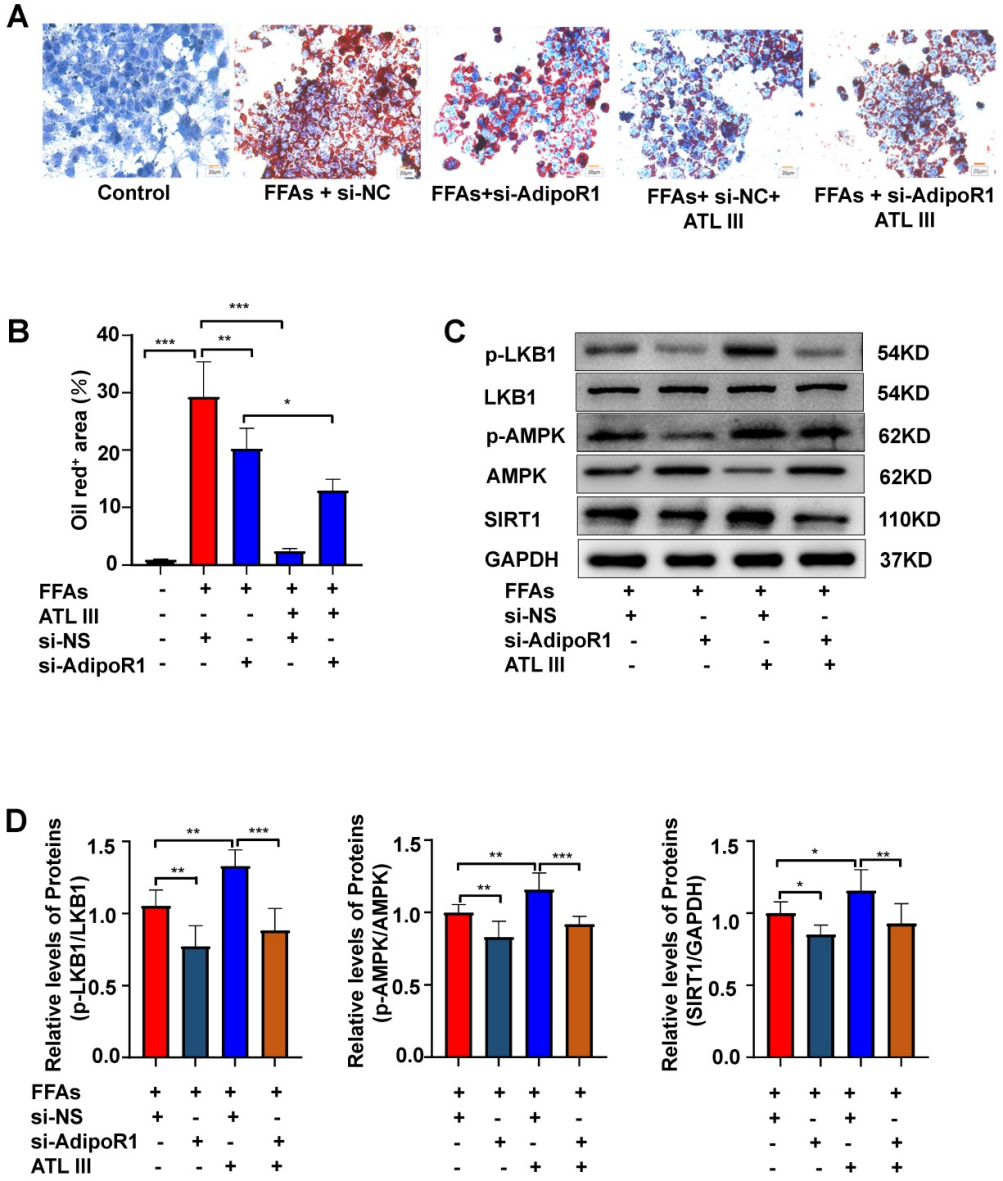
** Silencing AdipoR1 abolishes ATL III-mediated amelioration of lipid accumulation and AdipoR1 downstream signaling in HepG2 cells. A.** The expression of AdipoR1 was silenced by transfecting AdipoR1 siRNA into HepG2 cells. Lipids in HepG2 cells were stained by Oil Red O. Representative Oil Red O staining images were shown. **B.** The quantification of intracellular lipid content by Oil Red O staining was analyzed by calculating the area of intracellular lipid droplets. **C.** The expression levels of p-LKB1, p-AMPK and SIRT1 were examined by western blot. **D.** Relative levels of p-LKB1, p-AMPK and SIRT1 were analyzed. The experiments were performed four times independently, and values represent means±SD.*, *P*<0.05; **, *P*<0.01; ***, *P*<0.001.

To further confirm whether ATL III inhibits lipid deposition through AdipoR1 in hepatocytes, the expression of AdipoR1 was silenced by transfecting AdipoR1 siRNA into HepG2 cells. It was noted that the inhibition of lipid deposition induced by ATL III was blunted by the interruption of AdipoR1, suggesting that AdipoR1 plays a crucial role in ATL III mediated-inhibition of lipid accumulation (Figure 7A and 7B). In addition, the interruption of AdipoR1 suppressed ATL III-mediated up-regulation of p-LKB1, p-AMPK and SIRT1 in HepG2 cells (Figure 7C, 7D and Supplementary Figure S6).

### Inhibition of AMPK or SIRT1, the AdipoR1 downstream signaling, abolishes the protective effects of ATL III on lipid deposition and oxidative stress in FFAs-treated HepG2 cells

**Figure 8:**
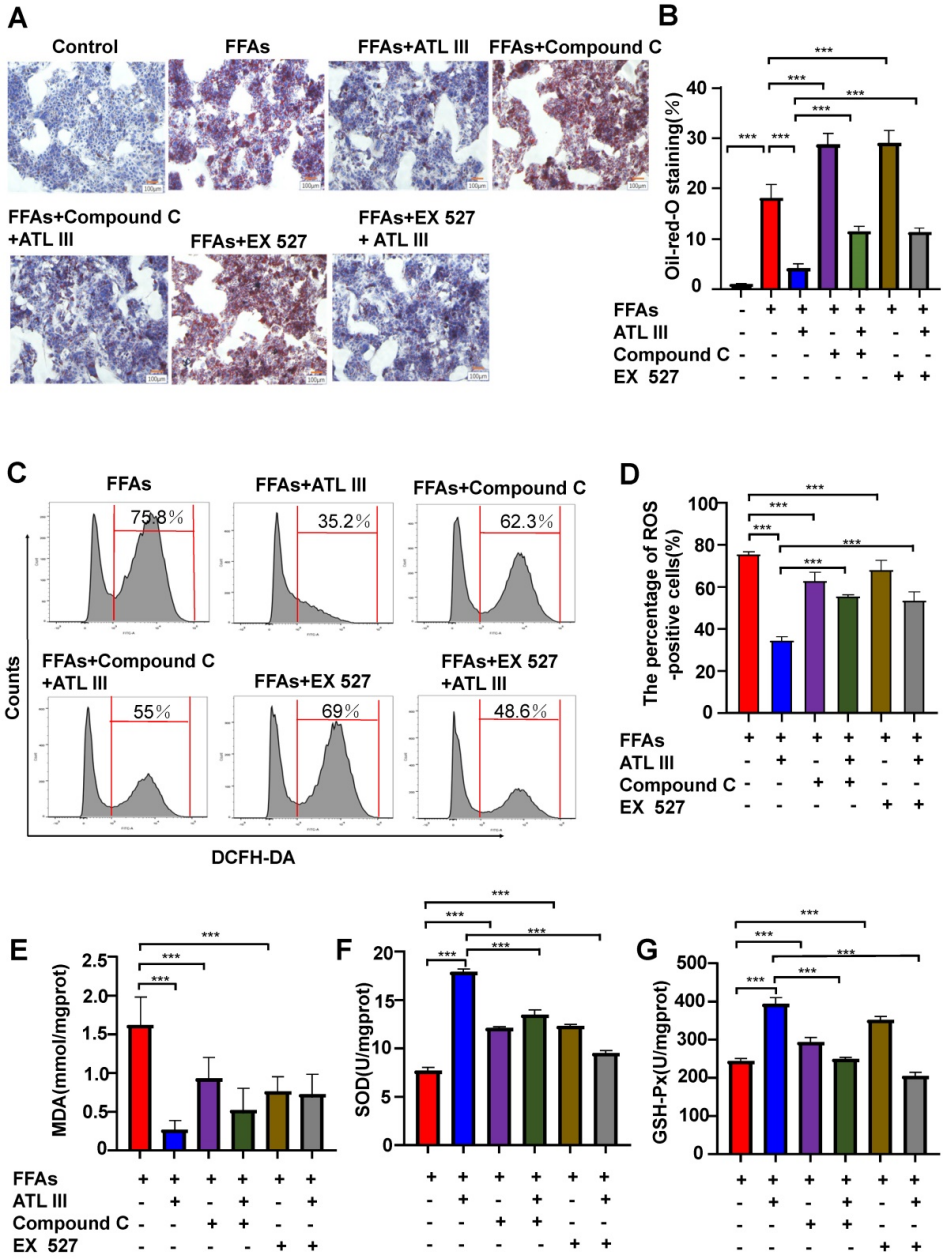
** Inhibition of AMPK or SIRT1 represses the inhibitory effects of ATL III on hepatic lipid deposition in FFAs-induced HepG2 cells.** HepG2 cells were categorized into seven groups including the control group, FFAs group, ATL III group, Compound C group, EX527 group, Compound C combined with ATL III group and EX527 combined with ATL III group. HepG2 cells were pre-incubated with Compound C (AMPK inhibitor) or EX527 (SITR1 inhibitor) for 1h, and then were incubated with FFAs for 24h, and were incubated with ATL III for another 24h. **A.** Lipids in HepG2 cells were stained by Oil Red O. Representative Oil Red O staining images were shown. **B.** The quantification of intracellular lipid content by Oil Red O staining was shown in the panel. **C.** ROS expression levels in HepG2 cells were tested by flow cytometry. Representative flow cytometry analysis of ROS levels in HepG2 cells were shown. **D**. The analysis of ROS levels expressed by HepG2 cells was shown. **E-G.** MDA, SOD and GSH-Px levels were measured. The experiments were performed three times independently, and the results were displayed as mean ± SD. *, *P*<0.05; **, *P*<0.01; ***, *P*<0.001.

The above results suggest that ATL III activates the AMPK and SIRT1 signaling pathways via the activation of AdipoR1. To investigate whether the AdipoR1 downstream AMPK and SIRT1 pathways contribute to the protective effects of ATL III, HepG2 cells were pre-treated with AMPK inhibitor (Compound C) or SIRT1 inhibitor (Ex 527) and then were treated with FFAs. As illustrated in Figure 8A and 8B, inhibition of AMPK or SIRT1 markedly enhanced FFAs-induced lipid accumulation. Importantly, the down-regulation of lipid accumulation induced by ATL III treatment was abolished by inhibiting AMPK or SIRT1.

Next, the effects of inhibitors of AMPK/ SIRT1 pathway on oxidative stress in FFAs-treated HepG2 cells were explored. It was not expected that the ROS levels in FFAs-treated HepG2 cells treated were higher than that in HepG2 cells treated by AMPK or SIRT1 inhibitors (Figure 8C and 8D). Crucially, the down-regulation of ROS levels induced by ATL III in FFAs-treated HepG2 cells was markedly blocked by inhibiting AMPK or SIRT1.

In addition, ATL III exposure markedly reduced FFAs-induced MDA levels in HepG2 cells, but the reduction was not obviously inhibited by co-treatment with AMPK or SIRT inhibitors (Figure 8E). Interestingly, the decreased GSH-Px and SOD levels in HepG2 cells induced by FFAs was partly abolished by co-treatment with AMPK or SIRT1 inhibitors. The up-regulation of GSH-Px and SOD levels induced by ATL III in FFAs-treated HepG2 cells were remarkably blocked after inhibition of AMPK or SIRT1 (Figure 8F and Figure 8G), which suggested that AMPK/SIRT1 pathway was involved in the elevation of GSH-Px and SOD levels regulated by ATL III.

### Inhibition of AMPK or SIRT1 abolishes the activated effects of ATL III on the SIRT1 downstream signaling in FFAs-treated HepG2 cells

**Figure 9:**
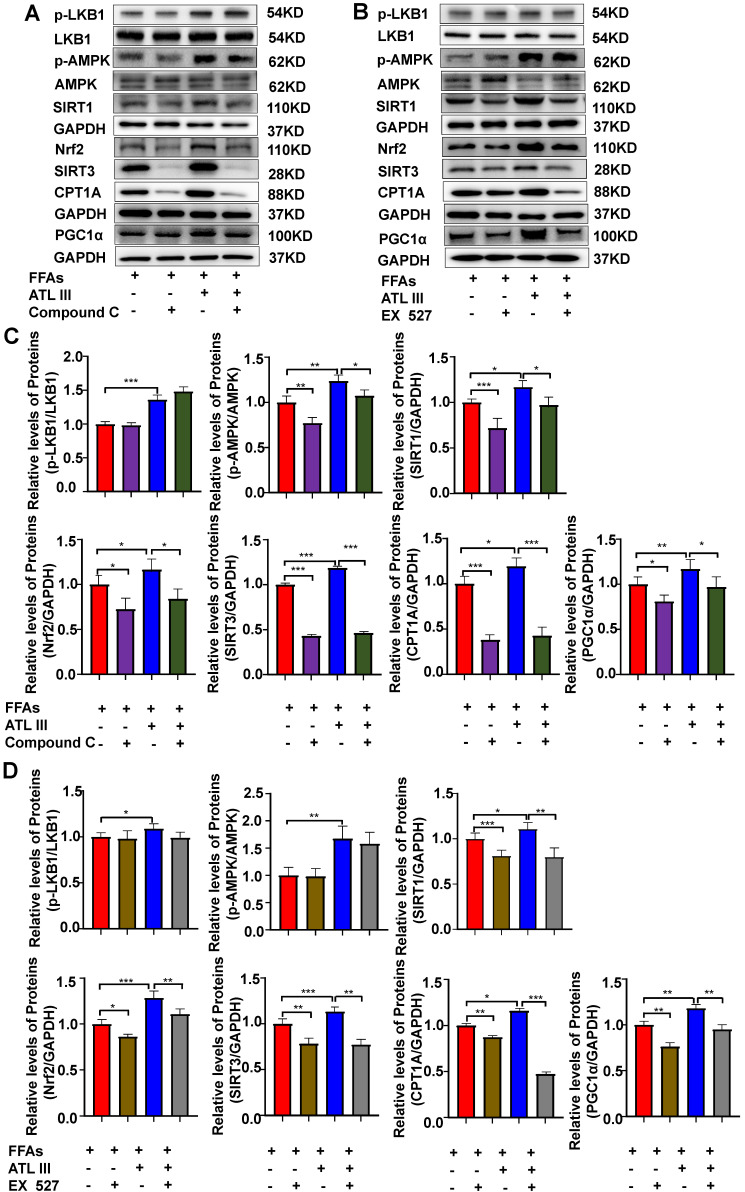
**Inhibition of AMPK or SIRT1 abolishes the ATL III-mediated activation of the SIRT1 downstream signaling in FFAs-treated HepG2 cells. A-B.** Levels of p-LKB1, p-AMPK, SIRT1, Nrf2, SIRT3, CPT1A and PGC1α protein were examined by western-blot. **C-D.** Relative levels of p-LKB1, p-AMPK, SIRT1, Nrf2, SIRT3, CPT1A and PGC1α were analyzed. The experiments were performed three times independently, and the results were displayed as mean±SD. *, *P*<0.05; **, *P*<0.01; ***, *P*<0.001.

To confirm the link between LKB1, AMPK and SIRT1, the protein levels of relevant signaling molecules in HepG2 cells were examined after inhibition of AMPK or SIRT1. As shown in Figure 9 and Supplementary Figure S7, the inhibition of AMPK or SIRT1 didn't affect the expression level of p-LKB1. The inhibition of AMPK reduced the up-regulation of SIRT1 induced by FFAs, but the inhibition of SIRT1 can't affect the up-regulation of p-AMPK. Further, the inhibition of AMPK or SIRT1 significantly reduced the protein levels of the SIRT1 downstream signaling pathways including Nrf2, SITR3, CPT1A and PGC1α. The above results reveal that AMPK acts as the primordial trigger for activating SIRT1 and its downstream signaling in FFAs-induced steatosis of HepG2 cells. Interestingly, the up-regulated expression of SIRT1 and its downstream signaling induced by ATL III in FFAs-treated HepG2 cells was partly blunted, which suggests that AMPK/SIRT1 signaling pathway is the crucial mechanism of ATL III ameliorating NAFLD.

## Discussion

NAFLD is a major health problem, which may occur in up to 20% of the general population worldwide 36. NAFLD is characterized by the accumulation of lipids in the liver arising from multiple factors: augmented fatty acid uptake, increased de novo lipogenesis, decreased fatty acid oxidation and very low density lipoproteins secretion. Therefore, a therapeutic option that targets multiple aspects in the pathogenesis of NAFLD is considered to be an effective strategy to treat this disease. The beneficial effects of traditional Chinese medicine on the treatment of NAFLD have been observed and widely accepted in recent years 37, however, the underlying mechanisms remain largely unknown. ATL III, the major bioactive component found in Atractylode smacrocephala Koidz, has been demonstrated to exhibit a series of benefits, including anti-oxidant, anti-tumor, anti-allergic response, anti-bacterial effects, and cognitive protection 17-19, but the effects of ATL III in NAFLD have not been explored. Our current study clearly revealed that ATL III treatment improved NAFLD in HFD-fed mice and FFAs-treated HepG2 cells. Especially, ATL III decreased liver inflammation and fibrosis induced by HFD to the levels equal or inferior to the ones observed in CD group, suggesting that ATL III has the therapeutical potential for the treatment of NAFLD.

One of the important findings in the current study is the discovery of AdipoR1 as a potential binding target for ATL III. AMPK signaling pathway is well known to play an essential role in controlling the development of NAFLD 9. Therefore, we focused on the genes being involved in this pathway to see if there is a candidate binding target of ATL III. The molecular docking based computational target fishing simulation was designed and performed in this study, and AdipoR1 and AdipoR2 were identified as the potential targets of ATL III. In addition, our data revealed that ATL III treatment restored hepatic expression of AdipoR1 that was markedly down-regulated in the HFD-induced NAFLD mouse model and FFAs-treated HepG2 cell model. Adiponectin is an adipocytokine that regulates glucose and lipid metabolism via the binding to its receptors AdipoR1 and AdipoR2, and the important roles of adiponectin and its receptors in regulating insulin resistance and lipid metabolism have been well documented 38, 39. The AdipoR1/AdipoR2 dual agonist has been shown to improve both NASH and fibrosis in mouse models 35. Therefore, ATL III may provide the pharmacological foundation for developing AdipoR1-based therapeutic agents on NAFLD.

Oxidative stress induced by free radicals plays an important role in the initiation and progression of NAFLD 40, 41. Accumulating studies suggest that hepatic MDA and ROS levels are significantly increased, while some anti-oxidative stress proteins including GSH-Px and SOD levels are significantly declined in NAFLD patients 42-45. Oxidative stress reflects an imbalance between bioavailability of ROS and the cellular antioxidant system that leads to a critical failure of cellular functions and eventually cell death 46. On the one hand, the levels of ROS and MDA were meaningfully increased in the HFD mouse model and FFAs-treated cell model, which were reduced by ATL III treatment. On the other hand, the levels of GSH-Px and SOD were decreased in the HFD mouse model and FFAs-treated cell model, which were up-regulated by ATL III. All these results suggest that ATL III treatment ameliorates hepatic steatosis by decreasing intracellular lipid droplets and oxidative stress.

To clarify the inhibitory effects of ATL III on lipid accumulation and oxidative stress, we investigated the mechanism of ATL III regulating lipid metabolism-related signaling pathways in HepG2 cells. The AMPK/SIRT1 signaling pathway is an important pathway involved in regulating hepatic lipogenesis, which is regarded as a therapeutic target for the treatment of NAFLD 47, 48. Our results showed that ATL III activated AMPK and SIRT1 signaling molecules, and further stimulated the downstream molecules including Nrf2, SIRT3, CPT1A, and PGC1α. When FFAs-treated HepG2 cells were treated with inhibitors of AMPK or SIRT1, the inhibitory effects of ATL III on lipid droplets in FFAs-treated HepG2 cells were blocked. In addition, inhibition of AMPK partly canceled out the increased protein levels of SIRT1 induced by FFAs, suggesting that AMPK was the downstream signaling molecule of SIRT1 activation induced by FFAs. Further, our data indicated that Nrf2, SIRT3, CPT1A and PGC1α were the downstream signaling molecules of SIRT1 because the activation of these pathways was suppressed by SIRT1 inhibitor. More importantly, the activation of AMPK /SITR1 signaling pathways induced by ATL III was partly blunted by AMPK inhibitor or SIRT1 inhibitor. Taken together, our results suggest that ATL III ameliorates NAFLD by activating multiple signaling pathways involved in lipid accumulation (LKB1 and AMPK), oxidative stress (SIRT3 and Nrf2) and fatty acid oxidation (CPT1A and PGC1α).

In summary, our results revealed that ATL III alleviates lipid accumulation and oxidative stress by promoting AdipoR1-mediated AMPK/SIRT1 pathways. Further investigation illustrates that this effect is mediated by regulating SIRT3, Nrf2, CPT1A and PGC1α expression. ATL III is a potential therapeutic candidate for the treatment of NAFLD.

## Supplementary Material

Supplementary figures.

Supplementary methods.

## Figures and Tables

**Figure 10:**
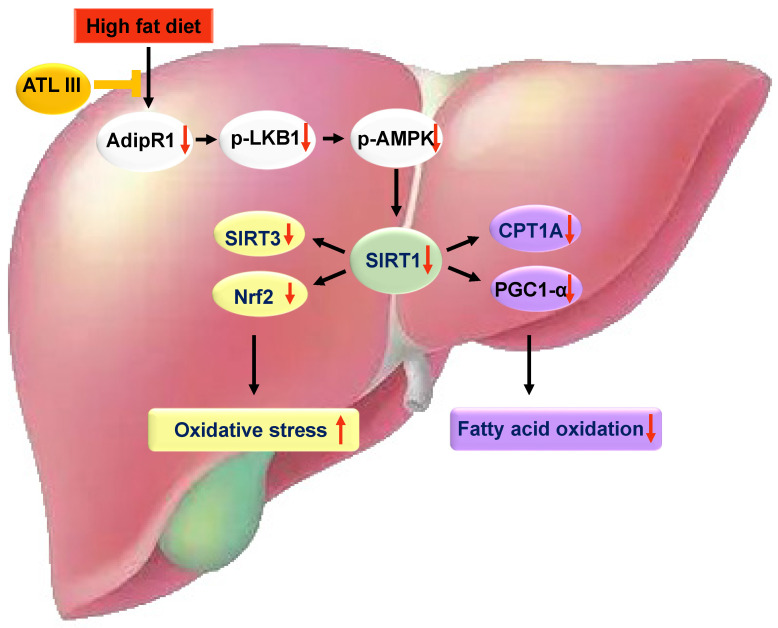
** A schematic overview depicting ATL III ameliorates NAFLD by activating the AdipoR1-mediated AMPK/SIRT1 pathway.** In both HFD-induced NAFLD mouse model and FFAs-induced HepG2 steatosis model, AdipoR1-mediated signaling pathway takes part in the protection roles of ATL III ameliorating NAFLD. Fatty liver induced by 3-month HFD feeding has prominent lipid accumulation with high levels of oxidative stress and fibrosis, which is inhibited by ATL III administration. In FFAs-induced HepG2 steatosis model, ATL III treatment activates AdipoR1-mediated AMPK/SIRT1 pathway. In one hand, ATL III increases SIRT3 and Nrf2 levels, which decreases oxidative stress. In another hand, ATL III increases PGC1 α and CPT1A levels, which contributes to fatty acid oxidation.

## Abbreviations

AdipoR1: adiponectin receptor protein 1
AdipoR2: adiponectin receptor protein 2
ADP: adenosine di-phosphate
ALT: alanine aminotransferase
AMP: adenosine mono-phosphate
AMPK: AMP-activated protein
AST: Glutamic oxaloacetic transaminase
ATL III: Atractylenolide III
BSA: bovine serum albumin
CCK-8: cell counting kit-8
CD: control diet group
IV-C: Collage type IV
CPT1A: carnitine palmitoyltransferase-1A
DCFH-DA: 2',7'-dichloro-dihydro-fluorescein diacetate
DMEM: Dulbecco's modified Eagle's medium
DMSO: dimethyl sulfoxide
FBS: fetal bovine serum
FFAs: free fatty acids
GSH-Px: glutathione peroxidase
HA: hyaluronic acid
HDL: high-density lipoprotein
HE: hematoxylin and eosin
HFD: high-fat diet
LDL: low-density lipoprotein
LKB1: liver kinase B1
LN: laminin
MDA: malondiadehyde
NAD+: nicotinamide adenine dinucleotide
NAFLD: nonalcoholic fatty liver disease
NASH: non-alcoholic steatohepatitis
Nrf2: nuclear factor erythroid-2-related factor 2
OA: oleic acid
PA: palmitic acid
PC III: procollagen type III
PGC1α: peroxisome proliferator-activated receptor gamma coactivator-1α
SIRT1: sirtuin 1
SIRT3: silent information regulator transcription 3
SOD: superoxide dismutase
TC: total cholesterol
TG: triglycerides
