# Supplementary material for: Atractylenolide III ameliorates Non-Alcoholic Fatty Liver Disease by activating Hepatic Adiponectin Receptor 1-Mediated AMPK Pathway
Source: Int J Biol Sci. 2022 Jan 31;18(4):1594–611. doi: 10.7150/ijbs.68873 (PMC8898375; doi:10.7150/ijbs.68873)
Supplement: Supplementary file 1 — Supplementary figures. [file ijbsv18p1594s1.pdf]

Supporting Materials

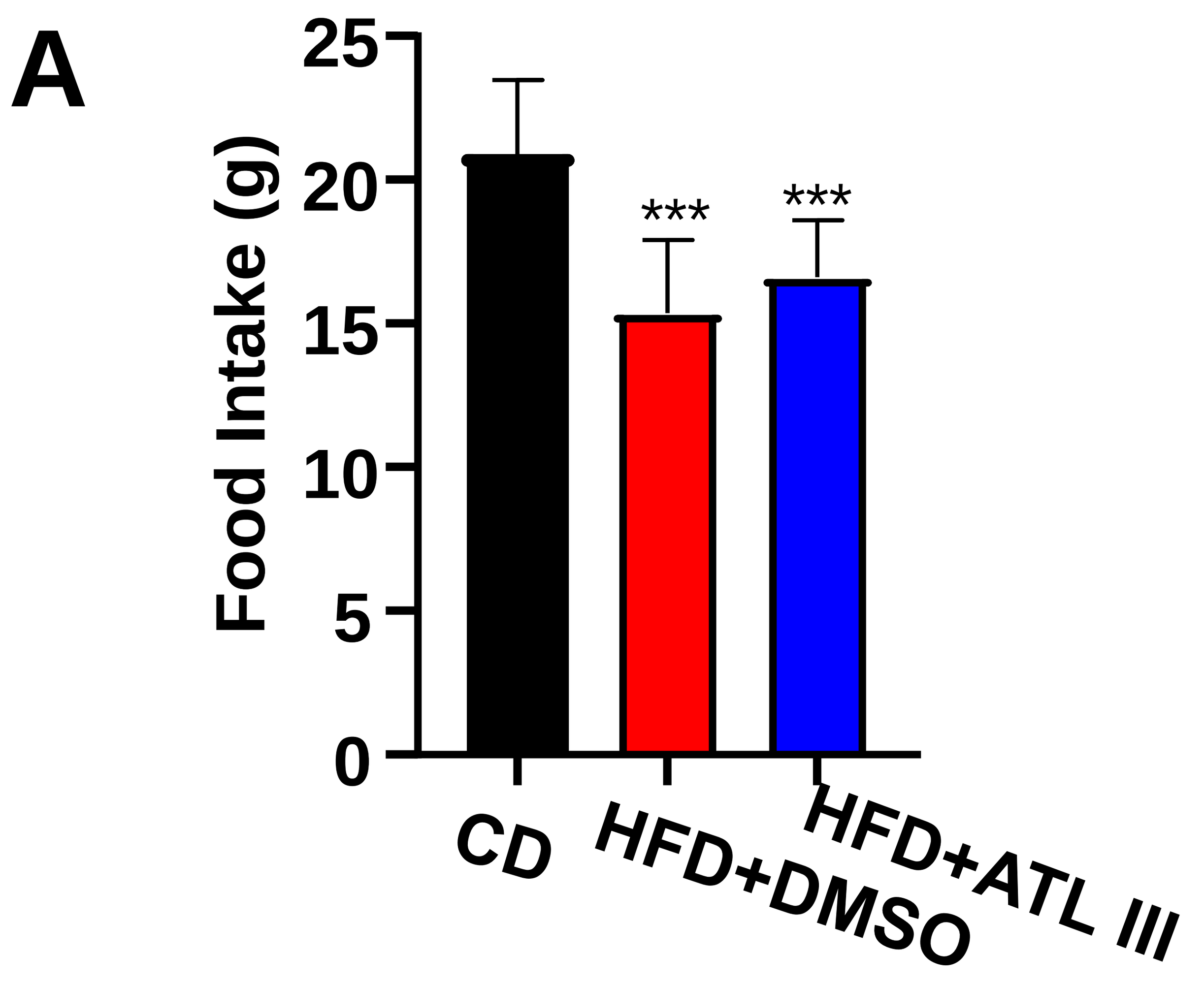

Supplementary Figure S1 ATL III didn't affect food intake after HFD feeding. A. Mice were categorized into three groups including control diet group (CD), the HFD-fed induced NAFLD mice group (HFD+DMSO) and the HFD-fed induced NAFLD mice group administrated with ATL III (HFD+ATL III). During the study, food intake per mouse per day was recorded and analyzed. *** , *P*<0.001.

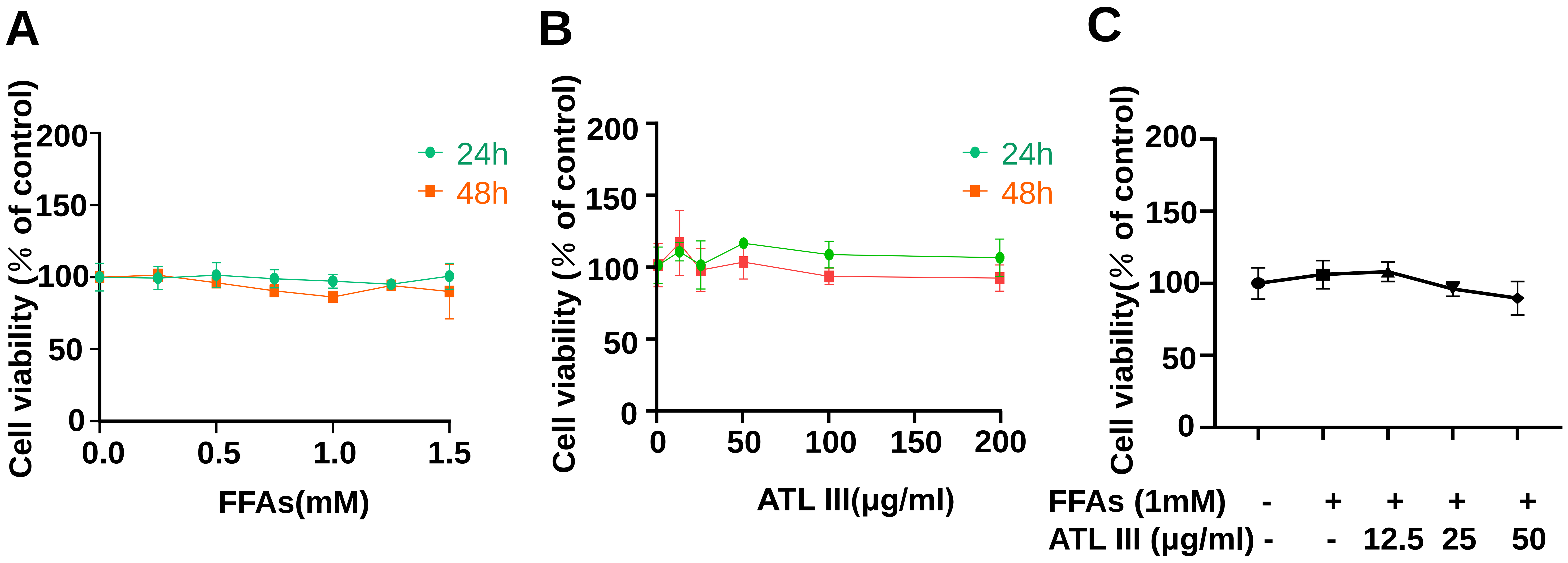

Supplementary Figure S2 ATL III administration didn't affect hepatocyte viability. HepG2 cells were treated with FFAs (0-1.5mM), ATL III (0-200ng/ml), or combination for 24 or 48 hours. **A-C**. The cell viabilities of HepG2 cells were measured. The experiments were performed three times independently., and the results were displayed as mean±SD.

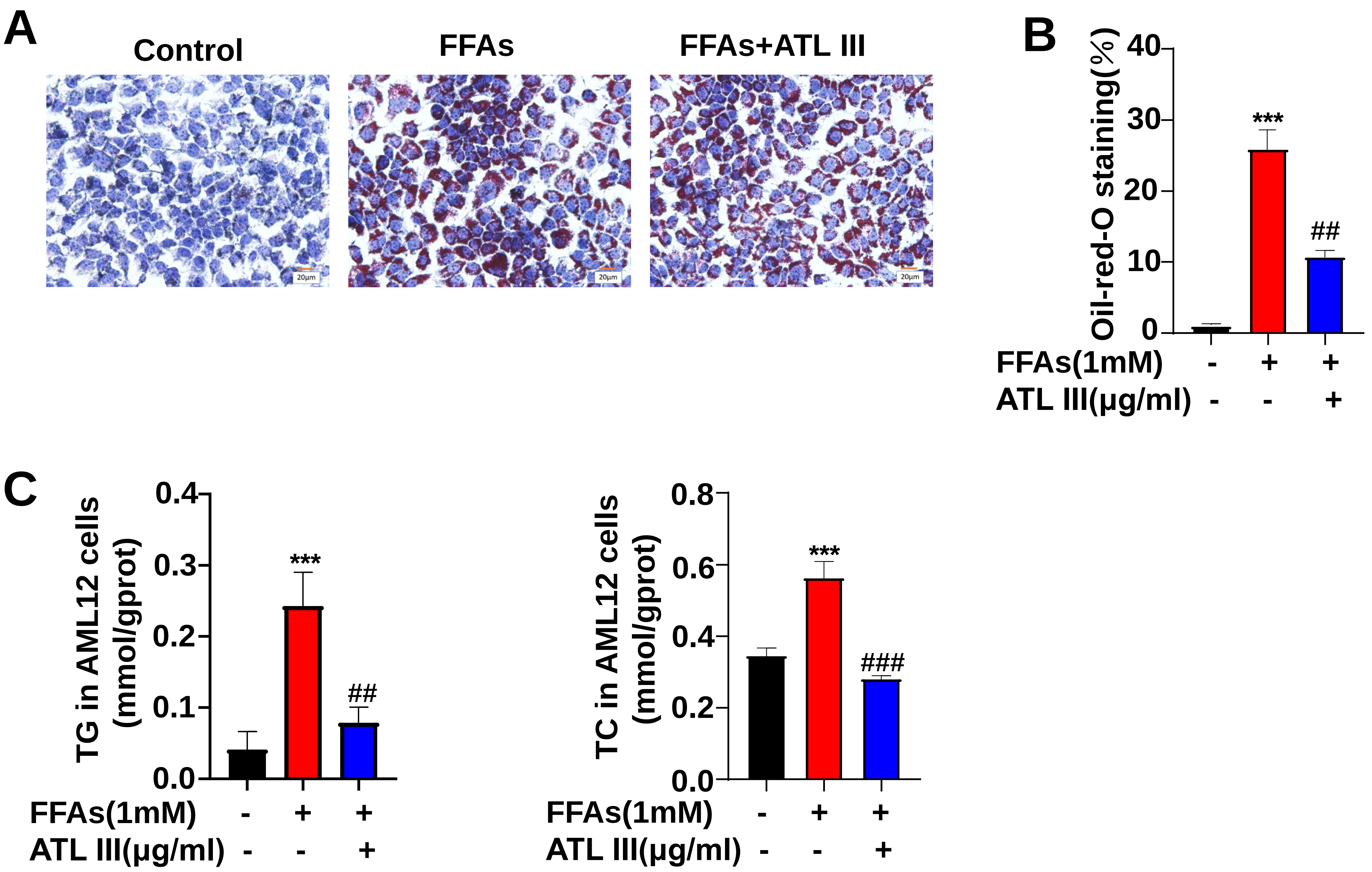

Supplementary Figure S3

AML12 cells were categorized into three groups including the control group (Control), FFA group (Model), 25 μg/ml ATL III group(ATL III group). A. Lipids in AML12 cells were stained by Oil Red O. Representative Oil Red O staining images are shown. B. The quantification of intracellular lipid content by Oil Red O staining is analyzed by calculating the area of intracellular lipid droplets. C. TG and TC levels were measured by biochemical test kits. The experiments were performed three times independently., and the results were displayed as mean ± SD. ***, compared with Control P<0.001; ##, compared with Model P<0.01; ###, compared with Model P<0.001.

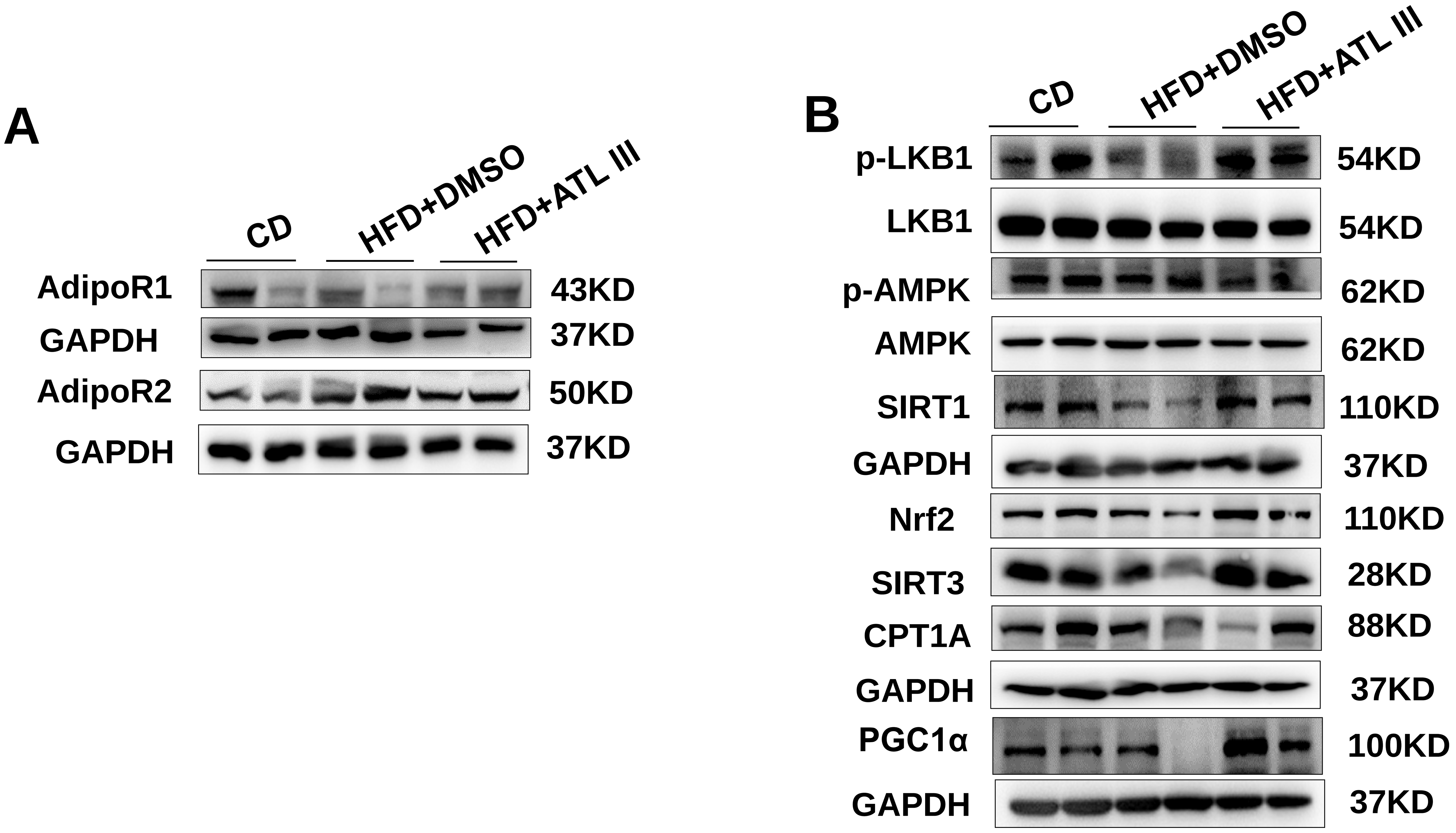

Supplementary Figure S4 ATL III treatment restores the down-regulation of hepatic AdipoR1 expression and AdipoR1 signaling induced by HFD feeding in mice. **A.** The results of repeated experiments concerning the expression levels of hepatic AdipoR1 and AdipoR2 in HFD mice. **B.** The results of repeated experiments concerning the expression levels of AdipoR1 signaling molecules in HFD mice.

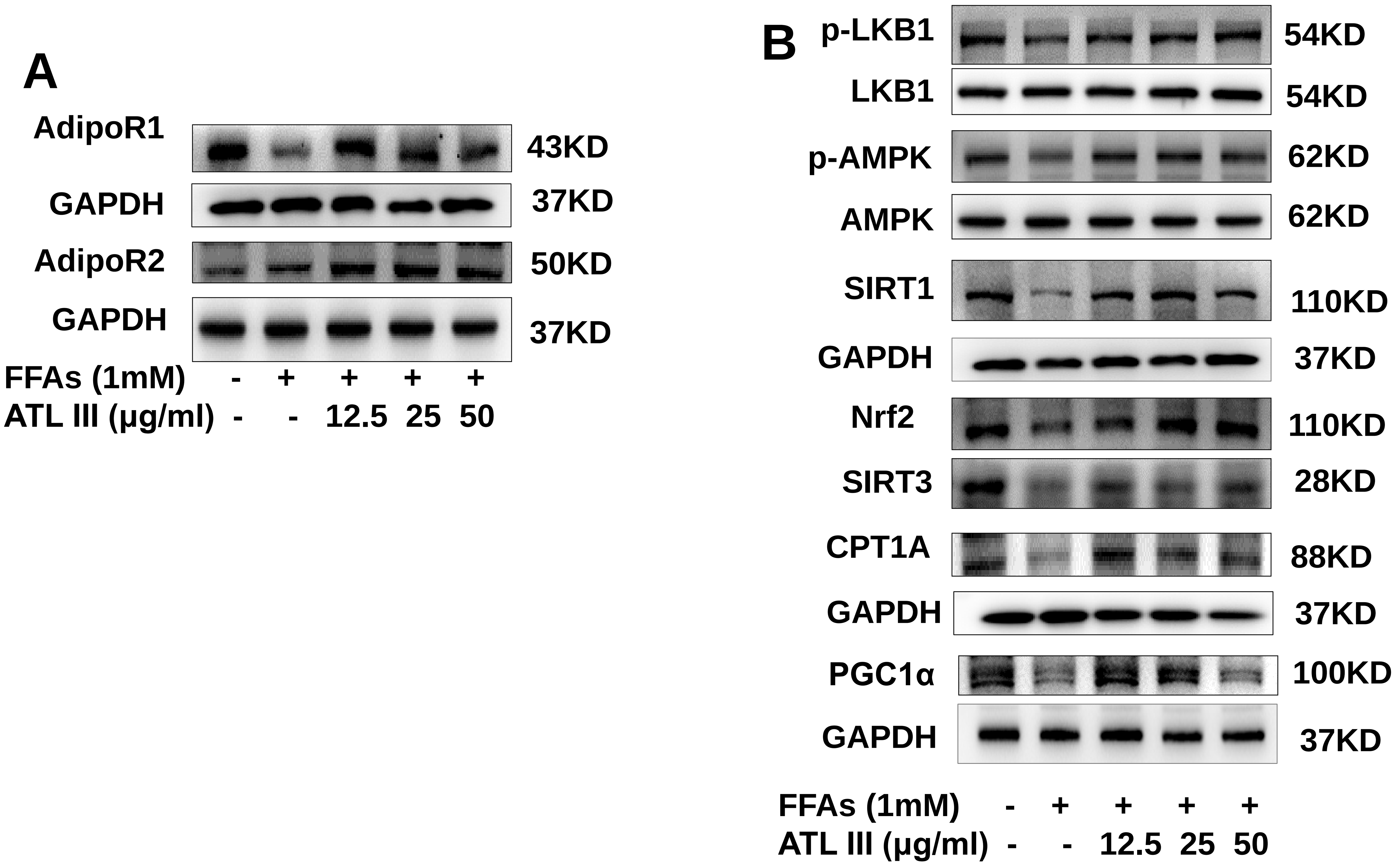

Supplementary Figure S5 ATL III restores the down-regulated expression of AdipoR1-mediated AMPK-SIRT1 signaling molecules in HepG2 cells . **A.** The results of repeated experiments concerning the expression levels of AdipoR1 and AdipoR2 in HepG2 cells. **B.** The results of repeated experiments concerning the expression levels of AdipoR1 signaling molecules in HepG2 cells.

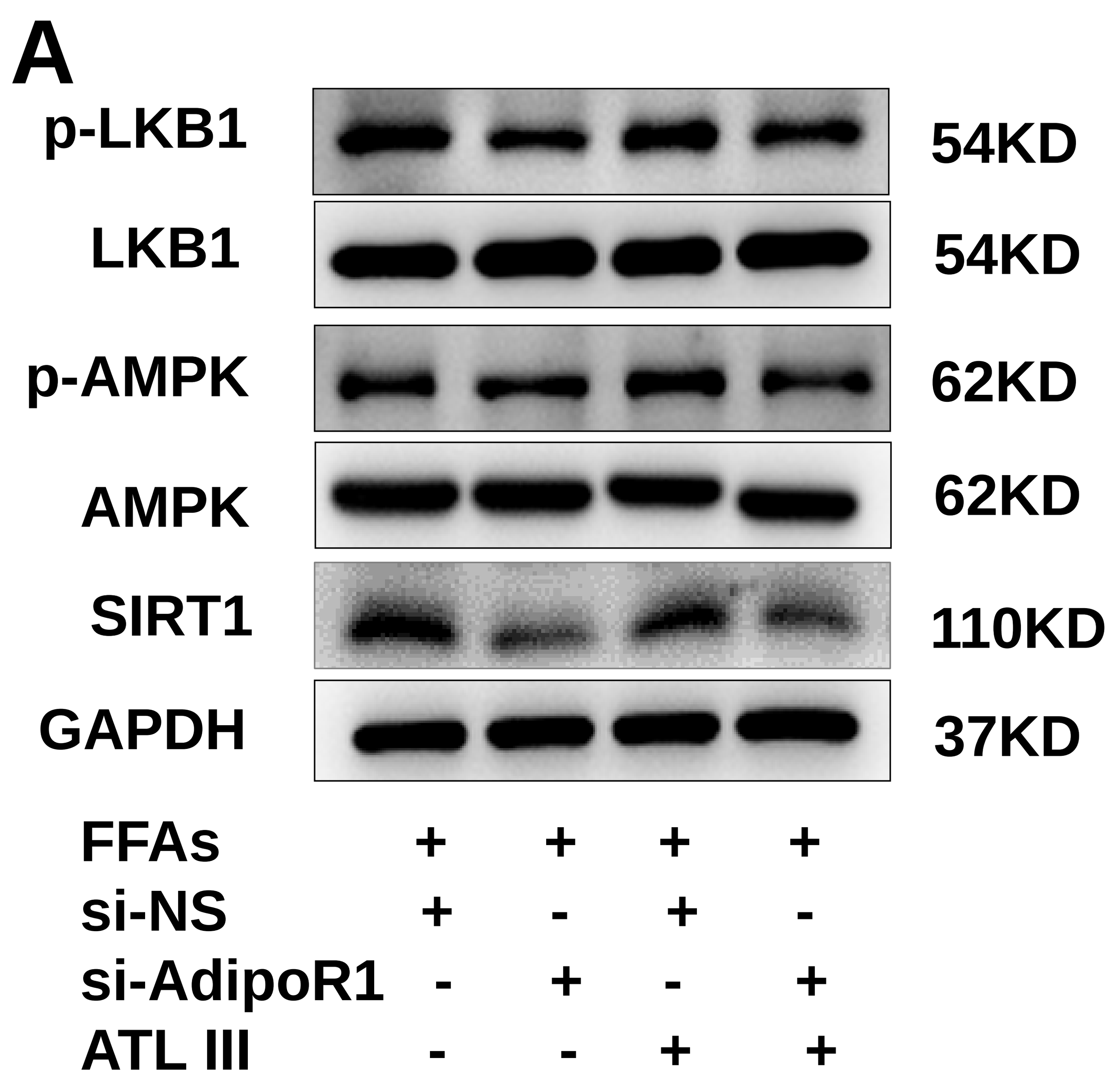

Supplementary Figure S6 Silencing AdipoR1 abolishes ATL III-mediated amelioration of lipid accumulation and AdipoR1 signaling in HepG2 cells . A. The results of repeated experiments concerning the expression levels of p-LKB1, p-AMPK and SIRT1 after silencing AdipoR1 in HepG2 cells.

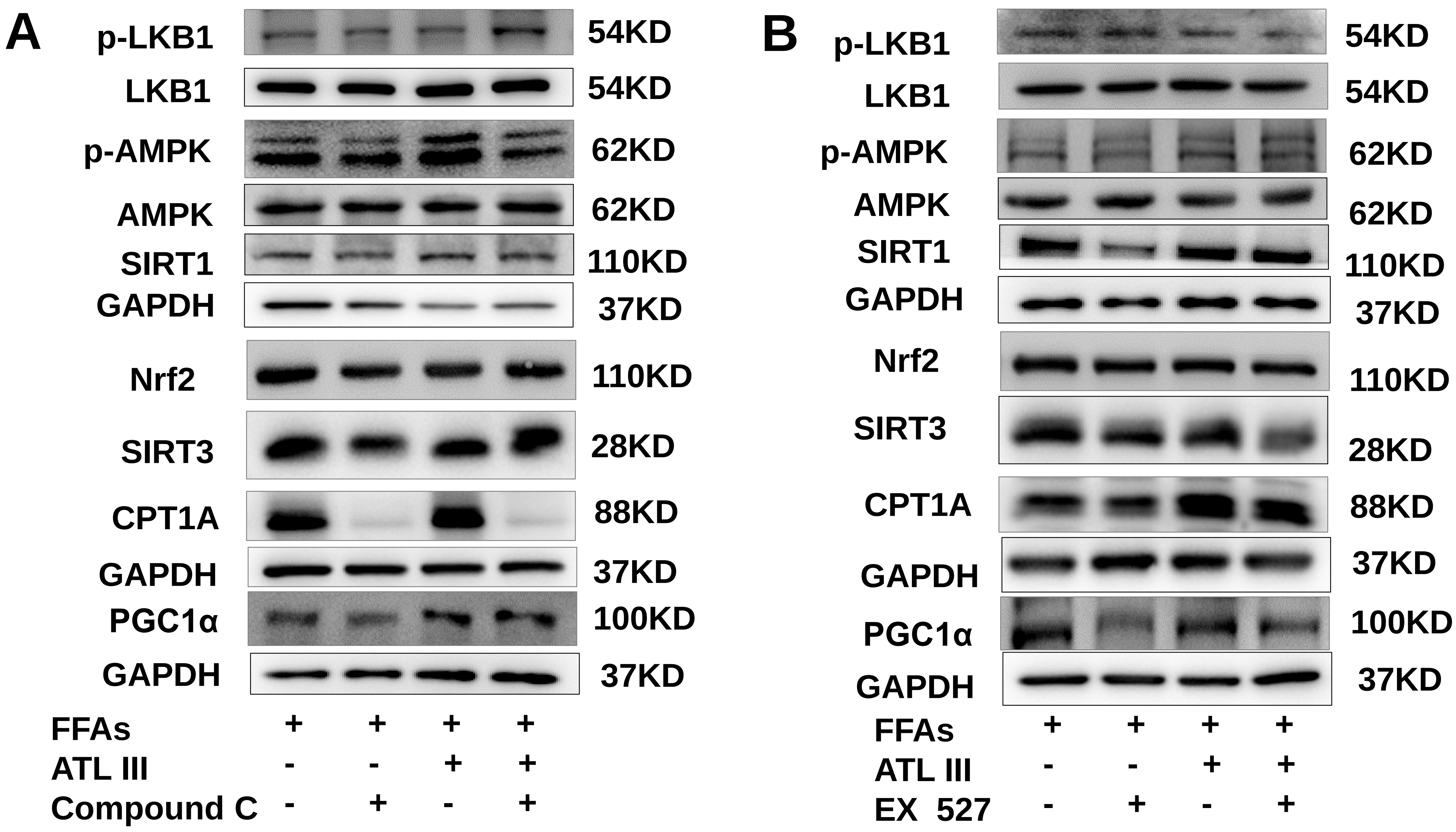

Supplementary Figure S7 Inhibition of AMPK and SIRT1 abolishes the ATL III-mediated activation of the SIRT1 downstream signaling in FFA-treated HepG2 cells A. The results of repeated experiments concerning the expression levels of AMPK downstream pathways in HepG2 cells after inhibition of AMPK. B The results of repeated experiments concerning the expression levels of SIRT1 downstream pathways in HepG2 cells after inhibition of SIRT1.
