# Supplementary material for: Atractylenolide III ameliorates Non-Alcoholic Fatty Liver Disease by activating Hepatic Adiponectin Receptor 1-Mediated AMPK Pathway
Source: Int J Biol Sci. 2022 Jan 31;18(4):1594–611. doi: 10.7150/ijbs.68873 (PMC8898375; doi:10.7150/ijbs.68873)
Supplement: Supplementary file 2 — Supplementary methods. [file ijbsv18p1594s2.pdf]

Supplementary methods

Quantitative real-time polymerase chain reaction (qRT-PCR)

Total RNAs were isolated from liver tissues of mice. For each sample, 1μg total RNA was used for cDNA synthesis (Vazyme Biotech Co, Ltd, China). The relative levels of mRNA to GAPDH were analyzed using an SYBR qPCR kit (Vazyme Biotech Co, Ltd, China). The qRT-PCR was performed on an ABI7500 RT-PCR system (Applied Biosystems, Foster City, CA). The sequences of primers were shown in Supplementary Table1. GAPDH was used as the normalized reference gene.

Supplementary Table 1 The primers used for qRT- PCR

Genes (mouse)	Forward primer(5’-3’)	Reverse primer(5’-3’)
TNF-α	AGGCTGCCCCGACTACGT	GACTTTCTCCTGGTATGAGATAGCAAA
IL-6	TCCATCCAGTTGCCTTCTTG	TTCCACGATTTCCCAGAGAAC
Col1α1	TAGGCCATTGTGTATGCAGC	ACATGTTCAGCTTTGTGGACC
Col3α1	TAGGACTGACCAAGGTGGCT	GGAACCTGGTTTCTTCTCACC
Col4α1	CCAGGATGCAACGGTACAAA	AACGTGGCCGAGAATTTCAC
α-SMA	TCCTGACGCTGAAGTATCCGATA	GGTGCCAGATCTTTTCCATGTC
Acc1	GATGAACCATCTCCGTTGGC	GACCCAATTATGAATCGGGAGTG

Srebp1c	GGCTCTGGAACAGACACTGG	TGGTTGTTGATGAGCTGGAG
Scd1	TTCTTGCGATACACTCTGGTGC	CGGGATTGAATGTTCTTGTCGT
GAPDH	TGTGTCCGTCGTGGATCTGA	CCTGCTTCACCACCTTCTTGA
